# Allograft inflammatory factor 1 is a potential diagnostic, immunological, and prognostic biomarker in pan-cancer

**DOI:** 10.18632/aging.204631

**Published:** 2023-04-03

**Authors:** Xin Liu, Dandan Zhang, Jianping Hu, Sikai Xu, Chengyun Xu, Yang Shen

**Affiliations:** 1 Department of Nephrology, The Second Affiliated Hospital of Nanchang University, Nanchang 330006, Jiangxi Province, China; 2 Department of General Surgery, The Second Affiliated Hospital of Nanchang University, Nanchang 330006, Jiangxi Province, China; 3 Jiangxi Key Laboratory of Molecular Medicine, The Second Affiliated Hospital of Nanchang University, Nanchang 330006, Jiangxi Province, China; 4 Department of Medical Genetics, The Second Affiliated Hospital of Nanchang University, Nanchang 330006, Jiangxi Province, China

**Keywords:** allograft inflammatory factor 1 (AIF-1), prognostic biomarker, immunotherapy response, KIRC, pan-cancer

## Abstract

Background: Allograft Inflammatory Factor 1 (AIF-1) is a member of the allograft inflammatory factor gene family and plays an essential role in the occurrence and development of malignant tumors. However, little is known about the expression pattern, predictive value, and biological function of AIF-1 across cancers.

Materials and Methods: We first analyzed AIF-1 expression across cancers based on data from public databases. Univariate Cox regression and Kaplan-Meier analyses were used to explore the predictive value of AIF-1 expression in various cancers. Moreover, gene set enrichment analysis (GSEA) was applied to determine the cancer hallmarks associated with AIF-1 expression. Spearman correlation analysis was performed to investigate the association between AIF-1 expression and tumor microenvironment scores, immune cell infiltration, immune-related genes, TMB, MSI, and DNA methyltransferases.

Results: AIF-1 expression was upregulated in most cancer types and exhibited prognosis-predictive ability. AIF-1 expression was positively correlated with immune infiltrating cells and immune checkpoint-related genes in most cancers. Additionally, the promoter methylation level of AIF-1 was different in distinct tumors. High methylation levels of AIF-1 were associated with a worse prognosis in UCEC and melanoma, whereas they were associated with a better prognosis in GBM, KIRC, OV, and UVM. Finally, we found that AIF-1 was significantly highly expressed in KIRC tissues. Functionally, silencing AIF-1 dramatically decreased proliferation, migration, and invasion abilities.

Conclusion: Our results reveal that AIF-1 acts as a robust tumor biomarker and is closely correlated with tumor immune infiltration. Furthermore, AIF-1 may function as an oncogene and promote tumor progression in KIRC.

## INTRODUCTION

Cancer is a global health problem that involves diverse genetic diseases. Over the past years, there has been an alarming increase in the occurrence of cancer, which continues to be the top cause of death globally [1]. Publicly available databases such as the Cancer Cell Line Encyclopedia (CCLE) and The Cancer Genome Atlas (TCGA) have been thoroughly analyzed and summarized to achieve a more comprehensive comprehension of the molecular mechanisms of human malignancy [2, 3]. With the continuous accumulation and development of multiomics data in various cancers, pan-cancer analysis has gained popularity as a research focus. Pan-cancer analysis has become a hot research direction [4, 5]. More importantly, unlike a study that analyzed a single and specific tumor type, pan-cancer research not only reveals similarities, heterogeneity, and breadth of analysis among different cancers but also provides a holistic overview of various aspects of cancer biology [6, 7].

Peptides generated by AIF-1 can bind calcium ions across lymphocytes, macrophages, and monocytes [8]. Therefore, the dysregulated AIF-1 expression has been strongly linked to a range of diseases, including cardiac allograft vasculopathy [9], rheumatoid arthritis [10], hemangioma [11], gastric cancer, glioma [12, 13], malignant breast tumor [14, 15], pancreatic carcinoma [16], and hepatocellular carcinoma [17]. Although the importance of AIF-1, its expression profile, prognostic significance, and functional implications in the majority of cancer types have yet to be systematically investigated. As such, there is a crucial need to take a fresh and comprehensive approach to examine the involvement of AIF-1 across different cancers.

In our analysis, we first analyzed the expression and gene mutation patterns of AIF-1 across cancers by integrating multiple databases. Subsequently, the clinical prognosis and functional analysis of AIF-1 were further explored. Furthermore, the link between AIF-1 expressions and specific studies, such as immune infiltration, immunological checkpoint genes (ICGs), tumor mutational burden (TMB), microsatellite instability (MSI), mismatch repair (MMR), and DNA methylation, was also thoroughly examined. Finally, AIF-1 was confirmed to be a strong oncogene in clinical specimens and *in vitro* experiments. AIF-1 reduction dramatically slowed the growth and occurrence of cancers. Therefore, the results of our pan-cancer analysis demonstrated that AIF-1 has the potential to be taken as a predictive biomarker for prognosis and immunotherapy response, which needs further investigation.

## MATERIALS AND METHODS

### Data collection

The UCSC Xena database (https://xenabrowser.net/datapages/) was taken to analyze the mRNA expression and clinical information of cancer patients in the TCGA cohort and the Genotype-Tissue Expression (GTEx) datasets. The transcriptomic profile of cancer cell lines was analyzed from the CCLE public database. The Clinical Proteomic Tumor Analysis Consortium (CPTAC) section of the University of ALabama at Birmingham CANcer data analysis Portal (UALCAN) (http://ualcan.path.uab.edu/index.html) was taken to explore AIF-1 expression profile in various cancers. In this study, we evaluated the protein expression profiling of AIF-1 in cancer patients with different grades, stages, ages, and weights. MMR gene mutation and DNA methylation analyses were also performed using the Sangerbox online platform. The correlation of five MMR genes or four methyltransferases with AIF-1 levels was evaluated by Spearman’s correlation method.

### Genomic variation analyses

The genomic alteration frequency, mutation type, mutation count, and copy number alteration of AIF-1 in the various cancer were investigated using the web tool cBioPortal (http://cbioportal.org) and are displayed in the module of “Cancer Types Summary” [18]. Additionally, the “Mutations” module presented the mutated site information of AIF-1 through a schematic or 3D representation of its protein structure. Using the “Comparison” module, we compared the overall survival, disease-free survival, progression-free survival, and disease-free survival differences between TCGA cancer cases with or without AIF-1 genetic alteration.

### Single-cell analysis of AIF-1

The Tumor Immune Single-cell Hub website (TISCH, http://tisch.comp-genomics.org/documentation/) utilized single-cell analysis to examine AIF-1 expression levels in different cell types, using major lineage as cell-type annotation and all cancers as cancer type. The results were presented through a heatmap and scatter diagrams. For more information on the data collection and steps, please refer to the documentation section on the TISCH website [19].

### Tumor immune infiltration analysis

To explore the relationship between AIF-1 expression and immune infiltrates in the pan-cancer, the “Immune-Gene” module of the TIMER2 web server was employed. Specifically, the study analyzed the correlations between AIF-1 mRNA expression and 21 different immune cell subsets, including CD4+ T cells, cancer-associated fibroblasts (CAFs), lymphoid progenitors, myeloid progenitors, monocyte progenitors, endothelial cells (Endos), eosinophils (Eos), hematopoietic stem cells (HSCs), T-cell follicular helper cells, γ/δ T cells, NK T cells, regulatory T cells (Tregs), B cells, neutrophils, monocytes, macrophages, dendritic cells, NK cells, mast cells, and CD8+ T cells. To estimate immune infiltration, various algorithms were employed. The purity-adjusted Spearman’s rank correlation test was used to calculate *P* and partial correlation (cor) values. The data were represented as a heatmap.

### AIF-1-related gene analysis

To investigate the protein-protein interaction network (PPI) of AIF-1, we utilized the GeneMANIA database to create the PPI network (http://www.genemania.org) [20]. Various bioinformatics approaches, such as physical interaction, coexpression, colocalization, gene enrichment analysis, and genetic interaction, were employed. Additionally, we examined the top 100 AIF-1-associated target genes from TCGA datasets of all tumors and normal tissues using the “correlation analysis” section of the GEPIA2 website. We used the “Gene_Corr” module to present the heatmap data of the target genes.

### Gene set enrichment analysis

The hallmark gene set, consisting of 50 gene sets, was obtained in the form of a “gmt” file (h.all.v7.4.symbols.gmt) from the Molecular Signatures Database (MSigDB, http://www.gsea-msigdb.org/gsea/index.jsp). We utilized this file to compute the normalized enrichment score (NES) and false discovery rate (FDR) of each biological process for each cancer type. GSEA analysis was performed with the R package “clusterProfiler,” and the findings were presented in a bubble plot generated using the “ggplot2” R package [21].

### Immunotherapy prediction analysis

The correlations between AIF-1 and immunotherapy biomarkers, including immune checkpoint genes (ICGs), tumor mutation burden (TMB), and microsatellite instability (MSI), were calculated using Spearman correlation analysis across various types of cancers [22, 23]. To investigate the correlation between ICGs, TMB, and MSI, we utilized Sangerbox (http://www.sangerbox.com/).

### Survival prognosis analysis of AIF-1

We utilized the survival analysis module available in GEPIA2 to generate a forest plot of overall survival (OS) and disease-specific survival (DSS) for AIF-1 across all TCGA tumors. The expression thresholds were set at a high cut-off value of 50% and a low cut-off value of 50% to partition the cohorts into high and low-expression groups. Additionally, we conducted a Kaplan-Meier curve analysis on the AIF-1 expression levels using the Sangerbox online platform.

### Epigenetic methylation analysis

To investigate the variation in methylation levels of AIF-1 between tumor and paired normal tissues across different TCGA cancer types, we employed the TCGA methylation module available in the UALCAN interactive web resource. We further examined the impact of methylation on prognoses by using the TIDE server, which can be accessed via the website http://tide.dfci.harvard.edu/.

### Cell lines, reagents, and plasmids

The Cell Bank of the Chinese Academy of Sciences (Shanghai, China) provided the 786-O cell line, a hyperdiploid renal cell carcinoma (RCC) cell line. The 786-O cell line was routinely maintained in Roswell Park Memorial Institute medium supplemented with 10% fetal bovine serum (Gibco, USA) at 37°C in a humidified atmosphere containing 5% CO_2_. A plasmid encoding shRNA against AIF-1 was synthesized by Genepharma Company (Shanghai, China). The cells were transfected with shRNA or vector plasmids using Lipofectamine 3000 (Invitrogen), following the guidelines provided by the manufacturer.

### Quantitative real-time (qRT)-PCR

Total RNA was isolated according to the Trizol Reagent protocol. The cDNA was synthesized from RNA using the PrimeScript RT Reagent Kit (Invitrogen) through reverse transcription. Subsequently, for qRT-PCR, the cDNA was amplified using the SYBR Green PCR Kit (Takara, China). Primer sequences were as follows: AIF-1 (5′-GTCCCTGAAACGAATGCT-3′ and 5′-GGAGCCACTGGACACCT-3′). GAPDH 5′-CCTGCCGGTGACTAACCCTG-3′ and 5′-AGTTAAAAGCAGCCCTGGTG-3′) were used as internal control.

### Cell proliferation and colony formation assays

Several cultures of 786-O cells were seeded onto 96-well plates at a density of 5.0 × 10^3 cells/well. The CCK-8 reagent (KeyGEN, Shanghai, China) was employed at specific time intervals to evaluate cell viability according to the manufacturer’s methods. Meanwhile, the Cell-Light EdU DNA Cell Proliferation Kit (RiboBio, Guangzhou, China) was used for the EdU assay. The cell proliferation rate of 786-O cells was determined by calculating the ratio of the EdU-positive cells to the total cell count. For the colony formation assay, cells transfected with the shRNA plasmid of AIF-1 were plated in 6-well plates at a density of 500 cells/well and cultured for 2 weeks. Then, the leaves were fixed and stained with 0.5% crystal violet.

### Western blotting

The exact quantity of protein added to each well of 12% SDS-PAGE gels. Briefly, the bands were blocked using 5% milk and then separated and transferred to membranes. The corresponding primary antibody was added to the membranes and then incubated overnight at 4°C. This step allows the antibody to specifically bind to the target molecule of interest on the membrane surface. Following this, the membranes were incubated with secondary antibodies, and the resulting bands were visualized using an ECL kit. Rabbit polyclonal anti-AIF-1 (10904-1-AP, Proteintech, China) and rabbit polyclonal anti-Tubulin (11224-1-AP, Proteintech) were purchased and used for western blot assays at diluted concentrations of 1:1,000 and 1:2,000, respectively.

### Transwell assay

Transwell systems (BD Biosciences, USA) with 8-μm pore size performed migration and invasion assays. In brief, RPMI-resuspended cells (5 × 10^4^) were seeded into the upper chambers of Transwell plates, either uncoated (for migration) or coated with Matrigel (for invasion). Medium containing 15% FBS was added to the lower chambers. After incubation for the specified duration, the chambers were taken out, and cells on the lower surface of the membrane were fixed, stained with 0.1% crystal violet, and photographed. Five random visual fields were manually counted for each chamber. Both migration and invasion assays were independently repeated three times.

### Statistical analysis

AIF-1 protein expression levels in clinical tumors and normal tissues were compared for statistical significance using a paired *t*-test. The predictive value of AIF-1 expression in each cancer was assessed using univariate Cox regression analysis and the Kaplan-Meier method. Correlations between groups were investigated using Spearman’s correlation analysis. Differences were explored using Student’s *t*-test or one-way analysis of variance. The significance level of *p* < 0.05 was used. The data underwent GraphPad Prism 9.0 software analysis and were repeated thrice. The findings are reported as the mean ± SD.

### Availability of data and materials

Publicly available datasets were analyzed in this study. The public databases included the TCGA database (https://gdc.cancer.gov/), GTEx database, THPA database (https://www.proteinatlas.org/), UCSC Xena database (https://xenabrowser.net/datapages/), and cBioPortal web (https://www.cbioportal.org/). The abbreviations of cancers are presented in Supplementary Table 1.

## RESULTS

### Basic information on AIF-1

**Figure 1:**
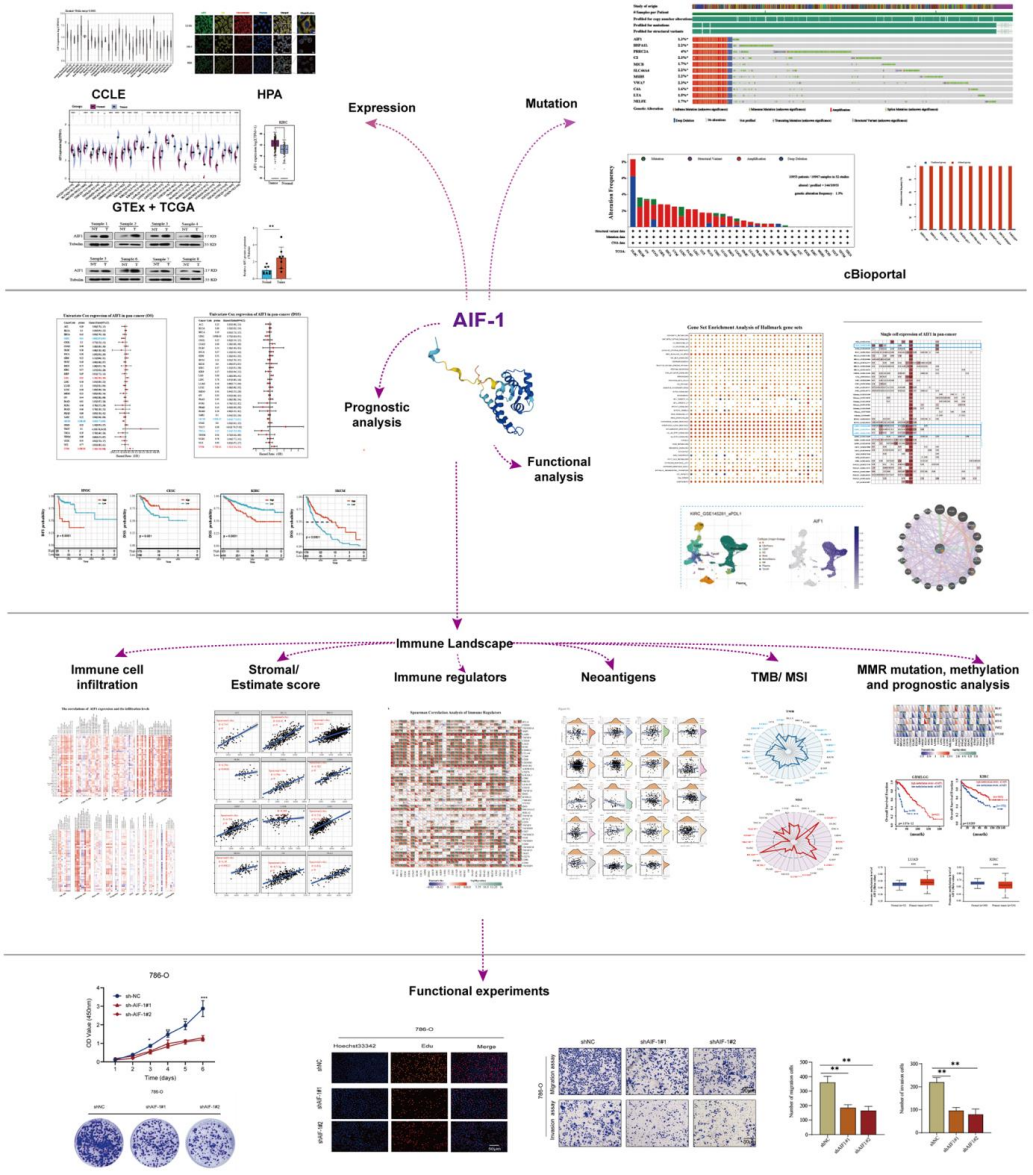
Flow chart of the entire study.

**Figure 2:**
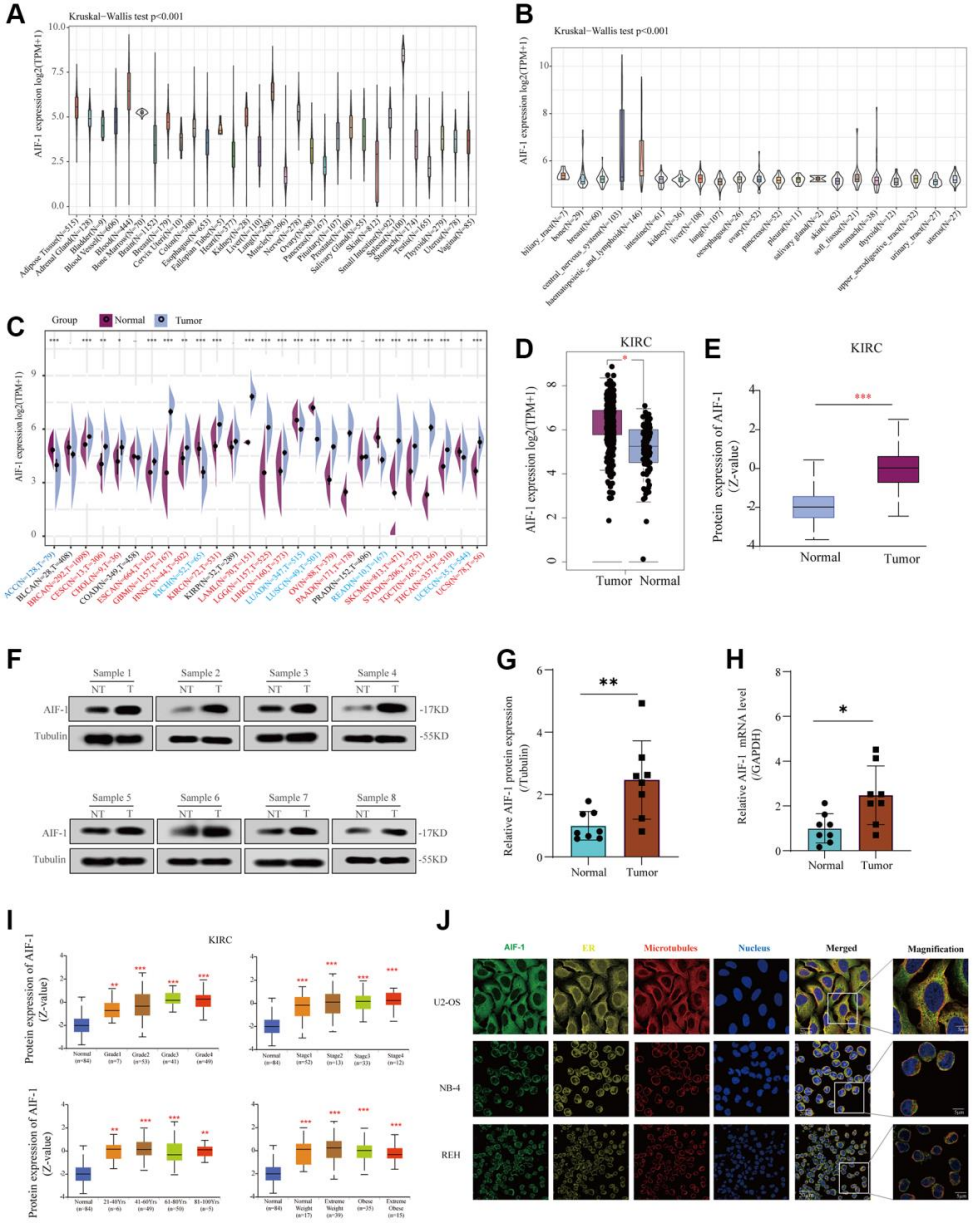
**Basic information of AIF-1.** (**A**) AIF-1 expression in 31 types of tissues. (**B**) AIF-1 expression in the cancer cell lines. (**C**) The level of AIF-1 expression between tumor and normal tissues in each type of cancer is based on the integrated data from TCGA and GTEx datasets. (**D**) The expression level of AIF-1 between KIRC and normal tissues. (**E**) The protein expression level of AIF-1 between tumor and normal tissues. (**F** and **G**) Determination and quantification of AIF-1 expression levels in KIRC tissues and paired normal tissues by western blotting assay. Tubulin was used as a loading control. (**H**) qRT-PCR analysis of AIF-1 mRNA expression in KIRC tumors and paired normal tissues. (**I**) AIF-1 expression levels in different pathologic grades, TNM stages, age phases, and body weights. (**J**) The immunofluorescence images of AIF-1 protein, nucleus, endoplasmic reticulum (ER), microtubules, and the incorporative images in U2-OS, NB-4, and REH cell lines. (^*^P < 0.05, ^**^P < 0.01, and ^***^P < 0.001).

The study incorporated samples from the TCGA database to be further analyzed. Figure 1 illustrates the flow chart of our study design. To examine the expression profile of AIF-1 across multiple tissues and cancer cell lines, we analyzed the mRNA expression levels of AIF-1 in 31 normal human organs (Figure 2A) and 21 cancer cell lines (Figure 2B) by utilizing data retrieved from the GTEx and CCLE datasets. Subsequently, the results showed that AIF-1 was highly expressed in BRCA, CESC, CHOL, ESCA, GBM, HNSC, KIRC, LAML, LGG, LIHC, OV, PAAD, SKCM, STAD, TGCT, THCA, and UCS. In contrast, ACC, KICH, LUAD, LUSC, READ, and UCEC exhibited low expression levels of AIF-1 (Figure 2C). Compared with normal tissues, the mRNA (Figure 2D) and protein (Figure 2E) expression of AIF-1 in KIRC was significantly upregulated. Thus, we further conducted western blot and qRT-PCR assays in clinical KIRC samples. As expected, the findings indicated that AIF-1 expression was significantly elevated in KIRC samples compared with adjacent tissues (Figure 2F–2H). We further assessed AIF-1 expression in cancer stages, pathologic grades, age phases, and body weight. As illustrated in Figure 2I, the AIF-1 expression was significantly increased in stages 1–4 of KIRC patients. We also found that those aged over 20 years old or overweight/obese/extremely obese with KIRC had higher expression levels. Finally, the immunofluorescence experiment presented that the AIF-1 protein was primarily distributed in the endoplasmic reticulum in the U2-OS, NB-4, and REH cell lines (Figure 2J).

### Mutation landscape of AIF-1 and enrichment analysis of AIF-1-related partners in pan-cancer

**Figure 3:**
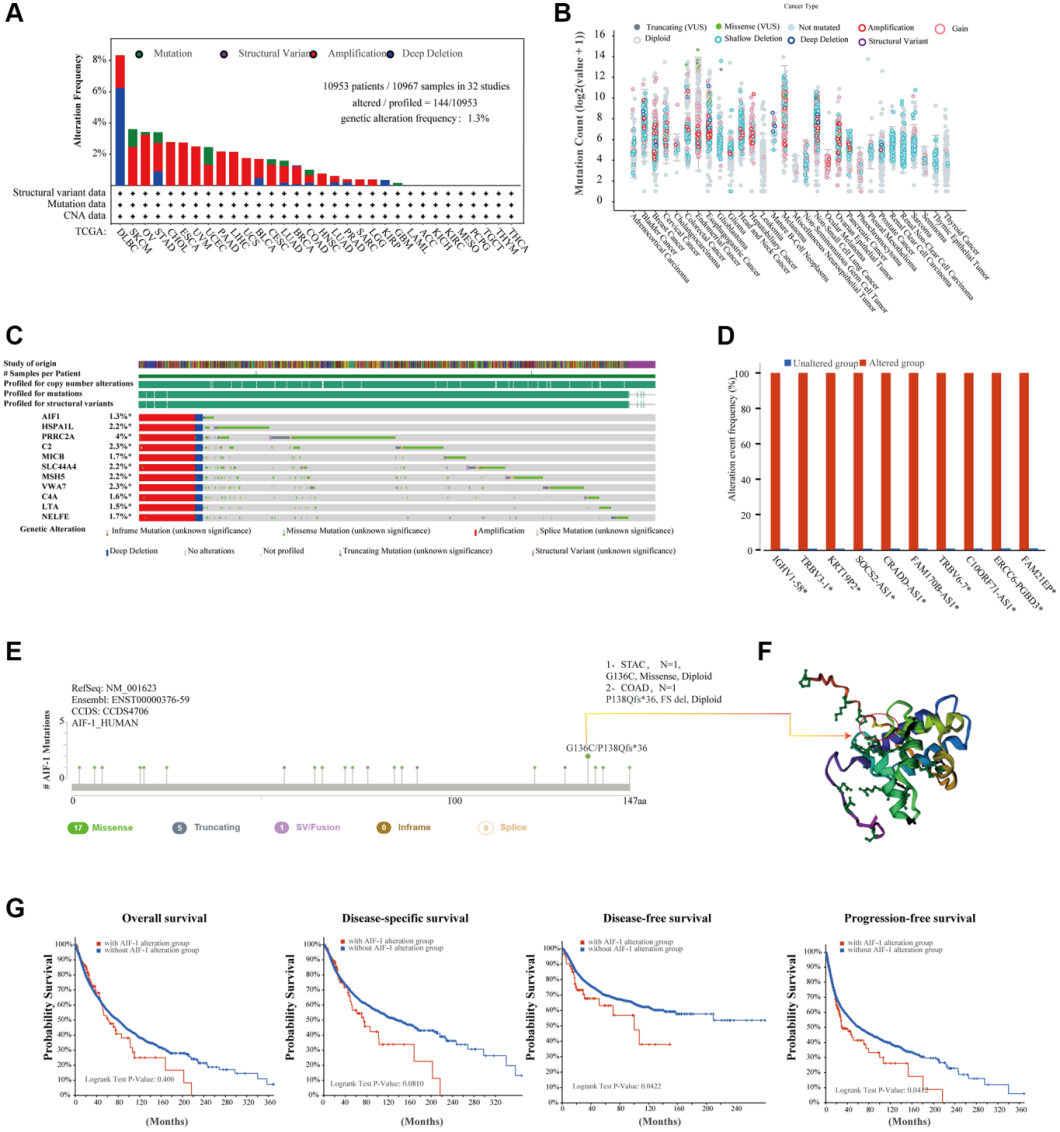
**Mutation landscape of AIF-1 in pan-cancer.** (**A**) The AIF-1 alteration frequency and mutation type in various cancers were displayed. (**B**) The entire mutation count of AIF-1 from the TCGA dataset is based on the cBioPortal tool. (**C**) Waterfall plot showing the cooccurrence pattern of AIF-1 alteration with genetic alterations of HSPA1L, PRRC2A, C2, MICB, SLC44A4, MSH5, VWA7, C4A, LTA, and NELFE. (**D**) Bar plot showing the frequencies of IGHV1-58, TRBV3-1, KRT19P2, SOCS2-AS1, CRADD-AS1, FAM170B-AS1, TRBV6-7, C10ORF71-AS1, ERCC6-PGBD3, and FAM21EP alteration cooccurrence with AIF-1 alteration. (**E**) Mutation sites are displayed in the AIF-1 structural domain. (**F**) The highest alteration frequency (G136C/P138Qfs*36) was displayed in the 3D structure of AIF-1 (labeled in yellow). (**G**) The potential correlation between AIF-1 mutation status and overall, disease-specific, disease-free, and progression-free survival. The original description lacked a comma between overall and disease specific survival.

We analyzed genetic alterations of AIF-1 in tumor samples from the TCGA pan-cancer cohort. As represented in Figure 3A, “Amplification” was the primary alteration type in most cancers. The highest AIF-1 alteration frequency (>8%) appeared in the patients with DLBC with “Deep Deletion” as the major alteration type. In addition, the mutation counts of each type in different cancers, including not mutated, deep deletion, missense, shallow deletion, truncating, gain in the frame, diploid, splice, amplification, and structural variant, are shown in Figure 3B. Furthermore, our analysis revealed a cooccurrence of genetic alterations between AIF-1 and other genes, as depicted in Figure 3C and 3D. These results suggested that these genes may act as functional partners in contributing to oncogenic effects AIF-1 across different cancer types. Furthermore, the sites and case numbers of AIF-1 alterations are shown in Figure 3E. The “missense” was the primary type of genetic alteration, and G136C was detected in STAC, which can induce a missense mutation and translation from glycine to cysteine. P138Qfs*36 was observed in patients with COAD, resulting in a frameshift or deletion mutation. Subsequently, the 3D structure of G136C/P138Qfs*36 in AIF-1 was observed, as shown in Figure 3F. Importantly, we investigated the possible correlation between AIF-1 genetic alterations and the clinical survival outcomes of the subjects. The results indicated that AIF-1 alteration showed a worse prognosis in disease-free survival (DFS) and progression-free survival (PFS) than patients without AIF-1 alteration. Nevertheless, there was no noteworthy distinction observed in the overall survival (OS) or disease-specific survival (DSS) (Figure 3G).

**Figure 4:**
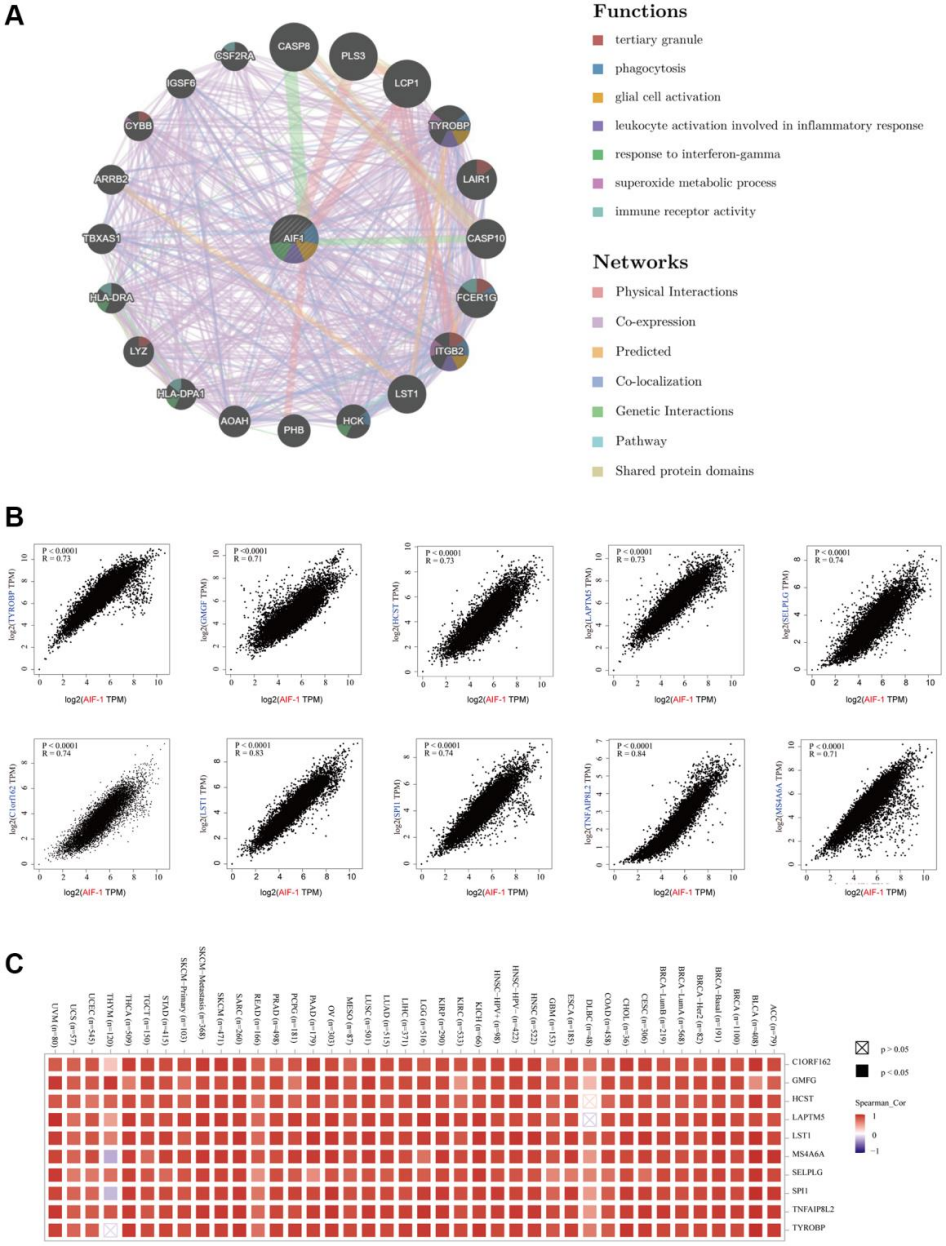
**Enrichment analysis of AIF-1-related partners.** (**A**) The GeneMANIA database showed the AIF-1-interacting gene network. The size of the node indicates the intensity of the connections. The internode connection lines represent gene-gene interactions, and the line color represents the types of interactions. The node color represents the possible functions of the respective genes. (**B**) The AIF-1-correlated genes in TCGA projects and the expression correlation between AIF-1 and the top 10 selected genes, including TYROBP, GMGF, LAPTM5, SELPLG, C1orf162, LST1, SPI1, TNFAIP8L2, and MS4A6A, were analyzed. (**C**) The corresponding heatmap data in the exact cancer types are displayed.

To unravel the potential molecular pathways by which AIF-1 contributes to cancer carcinogenesis, we created a PPI network for the AIF-1 gene by utilizing the GeneMANIA database in Figure 4A. The results showed that AIF-1 significantly interacted with CASP8, PLS3, LCP1, PHB, etc. We observed a positive correlation between the AIF-1 and TYROBP, GMGF, HCST, LAPTM5, SELPLG, C1orf162, LST1, SPI1, TNFAIP8L2, and MS4A6A genes (Figure 4B). The corresponding heatmap data showed a positive correlation between AIF-1 and the 10 genes mentioned above in most TCGA cancer types (Figure 4C).

### Single-cell analysis of AIF-1 in cancers

**Figure 5:**
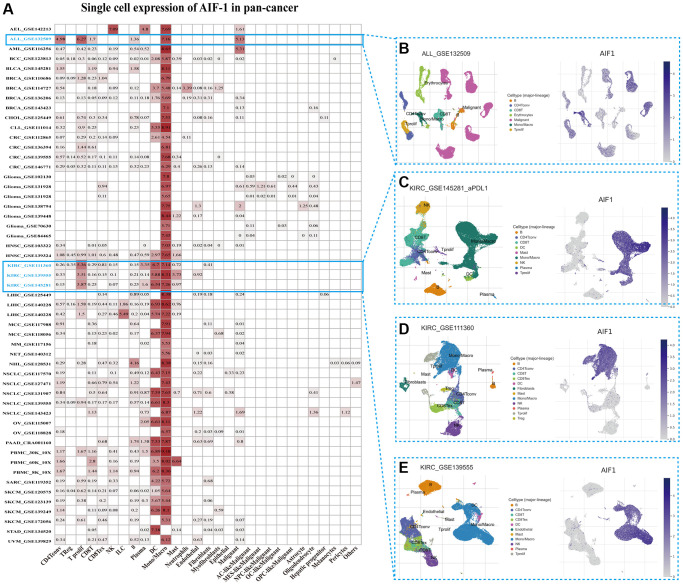
**Single-cell expression of AIF-1 across cancers.** (**A**) Summary of AIF-1 expression in various cell types in single-cell datasets. (**B**) Scatter plot showing the distributions of 7 different cell types in the GSE132509 ALL dataset. (**C**) Scatter plot showing the AIF-1 expression levels of cells in the GSE145281 KIRC dataset (**D**) Scatter plot showing the distributions of 12 different cell types in the GSE111360 KIRC dataset. (**E**) Scatter plot showing the distributions of 11 different cell types in the GSE139555 KIRC dataset.

To gain insight into the primary cell types expressing AIF-1 within tumor microenvironments, we conducted a single-cell analysis of AIF-1 in cancer sample datasets. The results indicated that (Figure 5A) AIF-1 was mainly expressed in monocytes/macrophages and malignant cells. The GSE132509 dataset, which contains 37,936 cells from 11 ALL patients treated with immune checkpoint inhibitors, was described in the immune cells types such as T cells, monocytes, macrophages, or malignant cells in the ALL microenvironment (Figure 5B). In the GSE145281 KIRC dataset with 44,220 cells and the GSE111360 KIRC dataset with 23,130 cells, AIF-1 was mainly expressed in malignant cells, monocytes, and macrophages in the KIRC microenvironment (Figure 5C and 5D). Furthermore, in the GSE139555 KIRC dataset with 49,907 cells, AIF-1 was highly expressed in DC and monocytes/macrophages cells (Figure 5E). The results showed that AIF-1 expression levels significantly increased in the mononuclear/macrophage cells of KIRC patients.

### TIMER immune cell infiltration analysis

**Figure 6:**
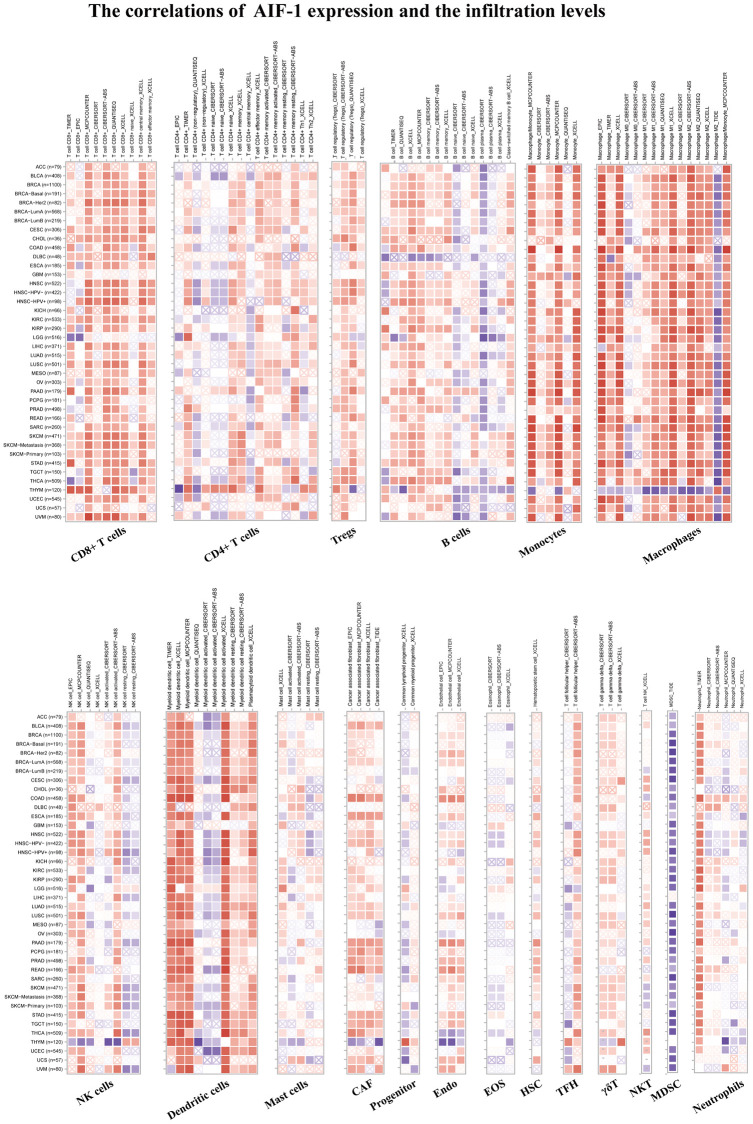
**TIMER immune cell infiltration analyses.** Correlations between AIF-1 expression and the infiltration levels of CD8+ T cells, CD4+ T cells, regulatory T cells (Tregs), B cells, monocytes, macrophages, NK cells, dendritic cells, mast cells, CAFs, progenitors, Endo, Eos, HSCs, TFH cells, γdT cells, NKT cells, MDSCs, and neutrophils in cancers. Positive correlation in red and negative correlation in blue.

We conducted further analysis to evaluate the connections between AIF-1 and cancer immunity by examining the correlations between AIF-1 expression and infiltration of immune cells. We revealed the landscape of AIF-1 associated with immune cell infiltration, including CD8+ T, CD4+ T, Tregs, B cells, monocytes, macrophages, dendritic, mast, CAFs, progenitors, Endo, HSC, Tfh cells, γ/δ T cells, NKT cells, MDSCs, neutrophils (Figure 6). AIF-1 was positively linked with the infiltration levels of CD8+ T cells, Tregs, monocytes, macrophages, and CAFs in most TCGA cancers. In contrast, AIF-1 expression was negatively related to the infiltration levels of MDSCs in most cancers, except for LIHC and THYM. In general, AIF-1 showed a positive correlation with the degree of immune infiltration exhibited by various types of infiltrating cells, including MDSCs, CD8+ T cells, macrophages, monocytes, and dendritic cells, in multiple cancers. In addition, we observed a significant correlation between the expression of AIF-1 and various types of infiltrating immune cells, including CD8+ T cells, CD4+ T cells, B cells, macrophages, NK cells, and dendritic cells in both thymic carcinoma (THYM) and thyroid carcinoma (THCA). Nonetheless, the nature of this correlation exhibited some variations, which could be attributed to the varying levels of immune infiltration in specific tumor types. Furthermore, ImmuneScore, EstimateScore, StromalScore, and neoantigens were integrated, suggesting that AIF-1 was associated with immune infiltration in some cancers (Supplementary Figures 1–4). Hence, these findings indicate that AIF-1 could potentially influence cancer’s progression, prognosis, and treatment through its interaction with immune cells.

### Associations between AIF-1 and immune regulators, TMB, and MSI

**Figure 7:**
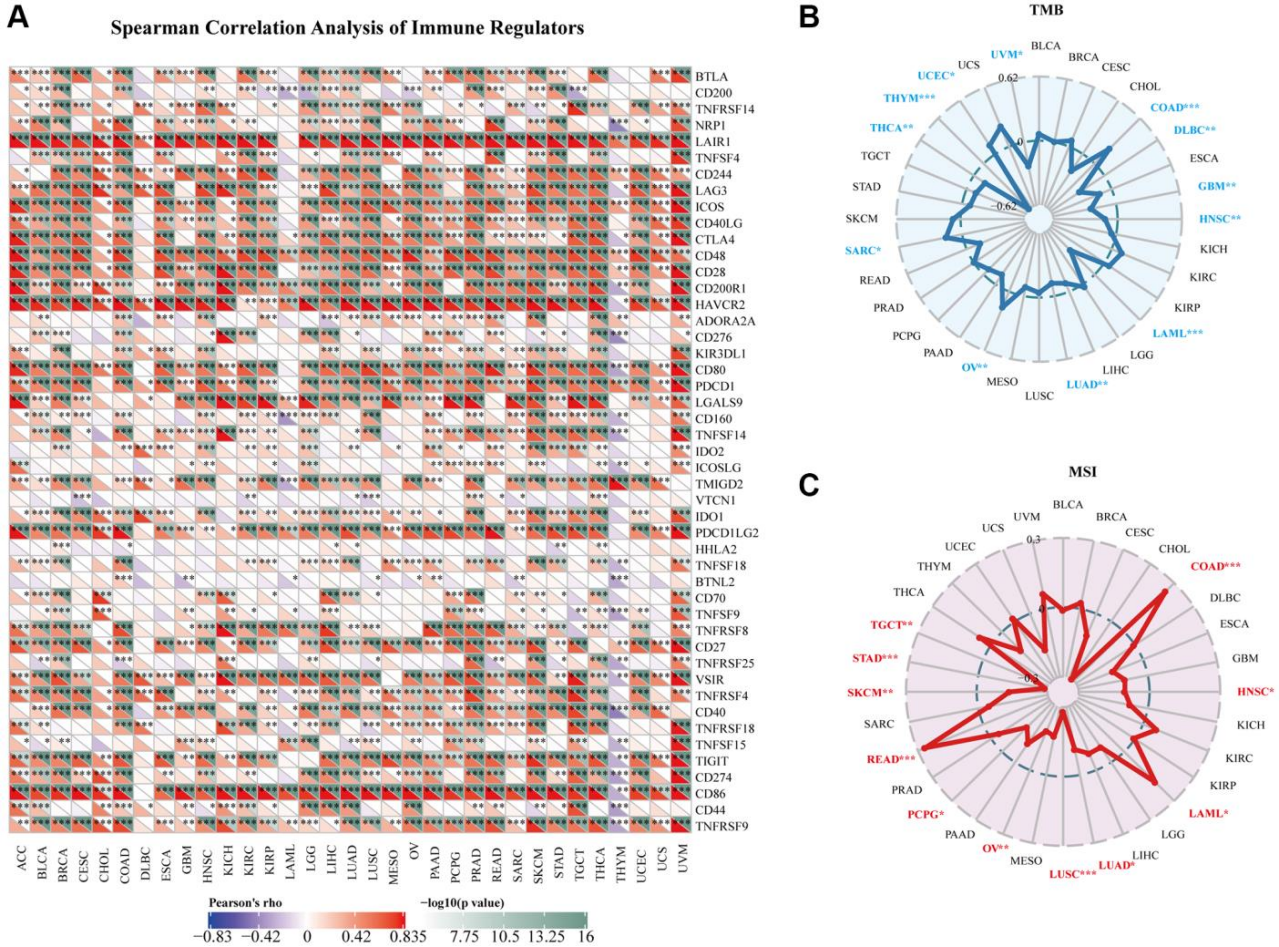
**Relationships between AIF-1 and immune regulators, TMB, and MSI.** (**A**) The Spearman correlation heatmap depicts the relationships between AIF-1 expression and the 47 different types of immune regulators in pan-cancer. Red color denotes a positive correlation, whereas blue color signifies a negative correlation. (**B**) Correlations between AIF-1 expression and tumor mutation burden (TMB) across cancers. (**C**) Correlations between AIF-1 expression and microsatellite instability (MSI) across cancers. (^*^P < 0.05, ^**^P < 0.01, and ^***^P < 0.001).

Figure 7A depicts the relationships between AIF-1 expression and the immune regulators in various cancers. In most cancers, a significant positive correlation was observed between AIF-1 and the majority of immune regulators, especially in ACC, BLCA, CESC, COAD, KIRC, LIHC, SKCM, TGCT, THCA, and UVM. Conversely, AIF-1 demonstrated a pronounced negative association with most immune regulators in THYM. In addition, a significant positive correlation observed between AIF-1 and several immune regulators, including LAIR1, CD244, LAG3, ICOS, CD48, CD28, HAVCR, CD80, PDCD1, CD27, VSIR, CD86, and TNFRSF9 in most cancers. To investigate the potential role of AIF-1 in predicting the efficacy of ICIs, we conducted additional analyses to evaluate the association between AIF-1 expression and TMB and MSI. Positive correlations with TMB were shown in UCEC, SARC, OV, and COAD. Negative correlations were observed in UVM, THYM, THCA, LUAD, LAML, HNSC, GBM, and DLBC (Figure 7B). Moreover, for the correlation between AIF-1 expression and MSI, positive associations were discovered in COAD, LAML, and READ. Negative correlations were found in HNSC, LUSC, LUAD, OV, PCPG, SKCM, STAD, and TGCT (Figure 7C). Based on our findings, it appears that AIF-1 may be able to serve as a predictor of the efficacy of immune checkpoint inhibitors (ICIs) in the specific types of cancers being studied.

### Prognostic analysis of AIF-1 in pan-cancer

**Figure 8:**
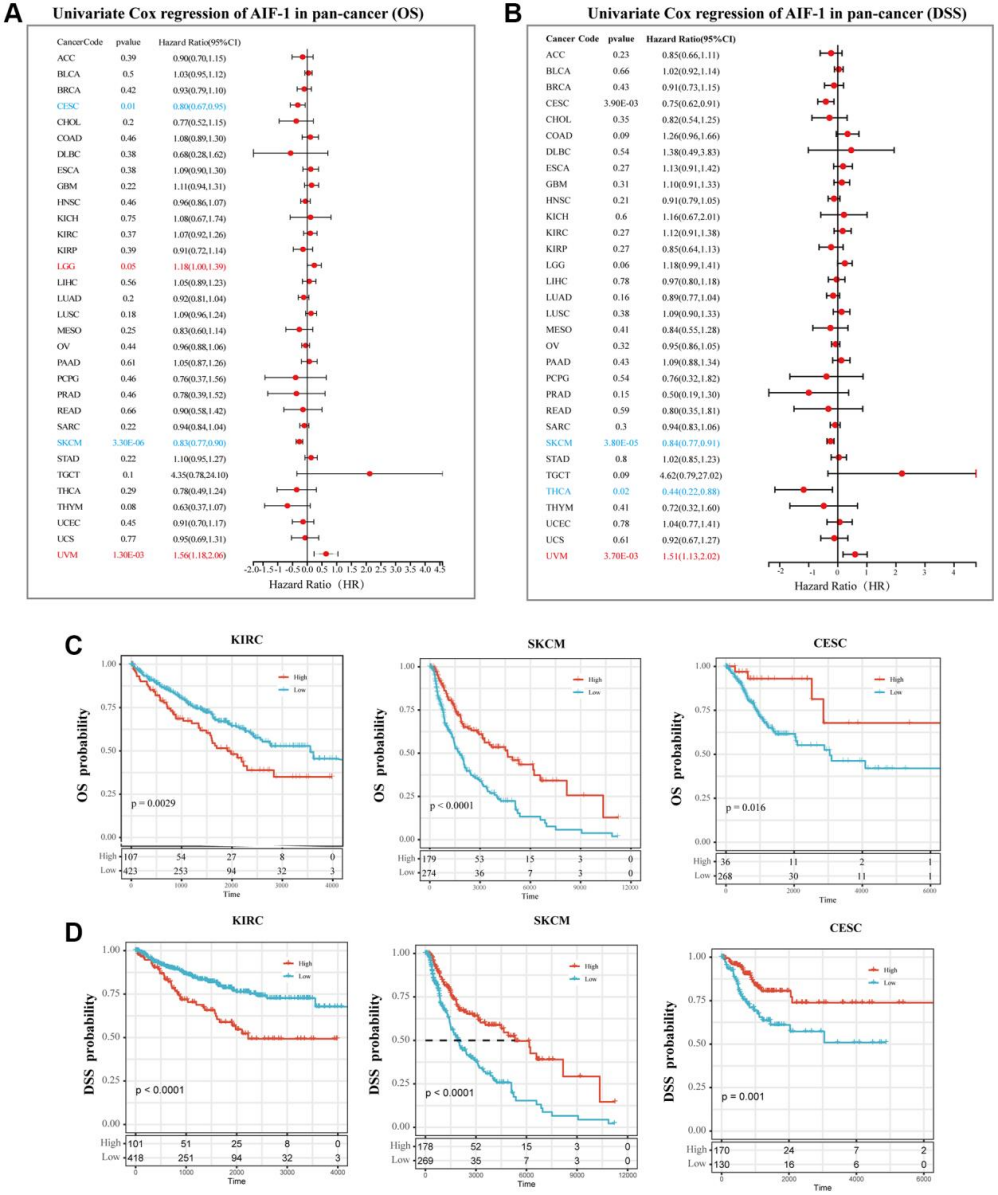
**Prognostic analysis of AIF-1 in pan-cancer.** (**A**) The forest plot shows the association between AIF-1 expression and OS by the univariate Cox regression method. (**B**) The forest plot shows the association between AIF-1 expression and cancer DSS by the univariate Cox regression method. (**C**) Kaplan-Meier OS curves of AIF-1 in CESC, KIRC, and SKCM. (**D**) Kaplan-Meier DSS curves of AIF-1 in CESC, KIRC, and SKCM.

We utilized the Kaplan-Meier method and univariate Cox regression to investigate the predictive potential of AIF-1 in the pan-cancer analysis. The forest plot results (Figure 8A) showed that downregulating AIF-1 had unique relationships with OS time prolongation in LGG (HR = 1.18 [95% CI, 1.00 to 1.39], *p* = 0.05) and UVM (HR = 1.56 [95% CI, 1.18 to 2.06], *p* = 0.0013). The overexpression of AIF-1 expression was related to the time delay of OS in CESC (HR = 0.80 [95% CI, 0.67 to 0.95], *p* = 0.01) and SKCM (HR = 0.83 [95% CI, 0.77 to 0.90], *p* < 0.0001). In addition, the relationships between AIF-1 expression and DSS in pan-cancer were also investigated. The results revealed a significant HR only in SKCM, THCA, and UVM (Figure 8B). In particular, SKCM had the most potent protective effect (HR = 0.84). Furthermore, the Kaplan–Meier curve analysis of KIRC CESC and SKCM showed that (Figure 8C) higher AIF-1 expression was associated with poor survival outcomes in KIRC and good survival outcomes in SKCM and CESC. Kaplan–Meier curve analysis for DSS in KIRC, CESC, and SKCM achieved consistent results (Figure 8D). The Kaplan-Meier curve analysis revealed that in KIRC, CESC, and SKCM, the expression of AIF-1 was associated with distinct survival outcomes. Specifically, higher expression of AIF-1 was linked to poor survival outcomes in KIRC. In contrast, in CESC and SKCM, higher expression of AIF-1 was associated with favorable survival outcomes. Consistent results were obtained from the Kaplan-Meier analysis of disease-specific survival (DSS) in KIRC, CESC, and SKCM, suggesting that AIF-1 may be a prognostic biomarker for these cancers. Figure 8C provides a visual representation of these findings. To further understand the prophetic role of AIF-1 in SKCM and UVM, univariate and multivariate Cox regression analyses were subsequently used to examine whether AIF-1 is an independent prognostic factor. The multivariate Cox regression analysis showed that AIF-1 was an independent protective factor for SKCM and an independent risk factor for UVM (Supplementary Figure 5).

### Correlation analysis with methylation profile

**Figure 9:**
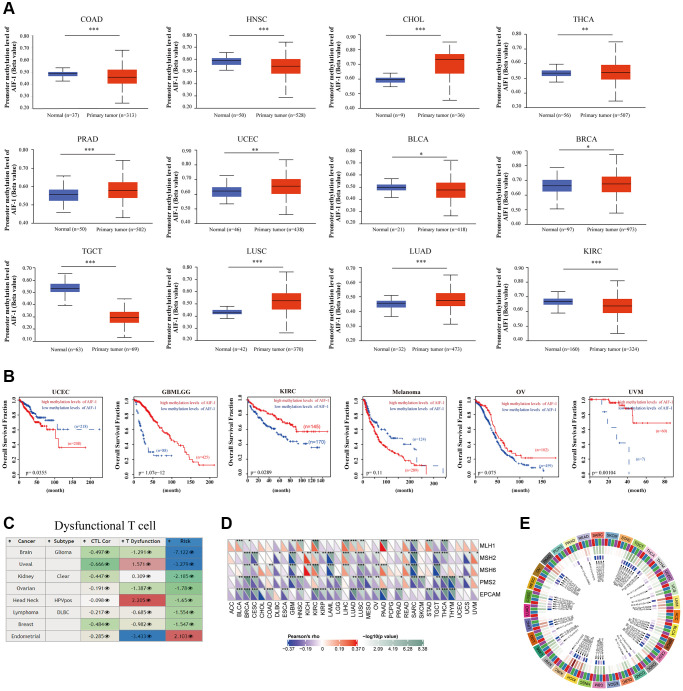
**Correlation analysis with methylation profile.** (**A**) Boxplots showing differential AIF-1 methylation levels (beta values) between tumors and adjacent tissues across the TCGA dataset. (**B**) Kaplan-Meier curves of OS differences between TCGA cancer cohorts with high methylation levels and those with low methylation levels of AIF-1. Only cancers with statistically significant differences between cohorts are presented. (**C**) Heatmap showing the roles of AIF-1 methylation in cytotoxic T-cell levels (CTLs), dysfunctional T-cell phenotypes, and risk factors in TCGA cancer cohorts. (**D**) Correlation between AIF-1 expression level and the expression of five MMR genes. The left bottom triangle in each unit denotes the coefficient of association calculated by Pearson’s correlation test. The top right triangle indicates the *P* value. (**E**) Correlation between AIF-1 expression level and four methyltransferase genes (DNMT1: red; DNMT2: blue; DNMT3A: green; DNMT3B: purple).

According to the findings, AIF-1 demonstrates hypermethylation in CHOL, THCA, PRAD, UCEC, BRCA, LUSC, and LUAD. However, it exhibits hypomethylation in several other cancer types, such as COAD, HNSC, TGCT, KIRC, and BLCA (Figure 9A). We discovered that hypomethylation of AIF-1 is correlated with shorter survival durations in GBMLGG, KIRC, OV, and UVM. Conversely, hypomethylation of AIF-1 is linked with a favorable prognosis in UCEC and melanoma (Figure 9B). In addition, our findings revealed a correlation between the methylation levels of AIF-1 and dysfunctional T-cell phenotypes in the glioma, uveal, ovarian, head and neck cancer, DLBC, breast cancer, and endometrial cancer cohorts (Figure 9C). Taken together, these results suggest that epigenetic methylation of AIF-1 in cancer patients is connected with dysfunctional T-cell phenotypes through various mechanisms, leading to distinct prognoses. Following our analysis of the association between AIF-1 methylation and prognosis, we investigated the relationship between AIF-1 expression and tumorigenesis mechanisms, specifically MMR defects and DNA methylation of crucial tumorigenesis genes. Our results revealed that AIF-1 was significantly correlated with the five MMR genes in pan-cancer (Figure 9D). Remarkably, most cancer types exhibited a negative correlation with these MMR genes, which could imply a potential role of MMR regulation in tumorigenesis. Additionally, we examined the expression of four methyltransferase genes (DNMT 1, 2, 3A, and 3B) in various cancer types and investigated their relationship with AIF-1. Our findings demonstrated coexpression between AIF-1 and these methyltransferase genes in almost all cancer types except for SKCM, PCPG, MESO, ESCA, DLBC, and ACC. The correlation coefficient was highest in TGCT, CESC, and KICH (Figure 9E).

### Gene set enrichment analysis of AIF-1

**Figure 10:**
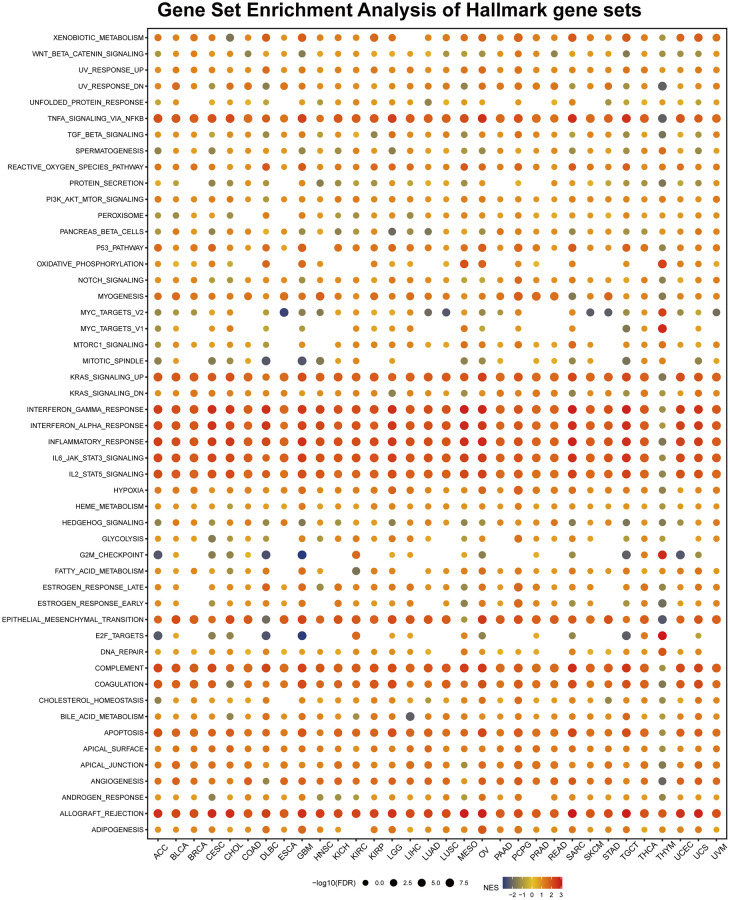
**Gene set enrichment analysis (GSEA) of AIF-1 in pan-cancer.** The circle size represents the FDR value of the enriched term in each cancer, and the color indicates each term’s normalized enrichment score (NES).

To identify the cancer hallmarks associated with AIF-1 expression, we used differentially expressed genes (DEGs) between low-AIF-1 and high-AIF-1 subgroups in each cancer to conduct GSEA. This enabled them to determine which cancer hallmarks were enriched in the low- or high-AIF-1 subgroups, providing insight into the molecular mechanisms underlying cancer development and progression. The study revealed a significant correlation between AIF-1 expression and various immune-related pathways, including but not limited to TNFA signaling via NFKB, KRAS signaling, IL6-JAK-STAT3 signaling, IL2-STAT5 signaling, epithelial–mesenchymal transition, compliment, and allograft rejection pathways (Figure 10). The data obtained from the study suggest a possible correlation between AIF-1 expression and immune activation in the tumor microenvironment (TME). In addition, the IFN-a response, IFN-g response, and EMT were significantly enriched in the high-AIF-1 subgroup of all cancers.

Earlier research has demonstrated that epithelial-mesenchymal transition (EMT) is associated with cancer onset, spread, and drug resistance [24], implying that AIF-1 may have a crucial role in the formation and metastasis of cancer by participating in the EMT process. To summarize, the study’s results suggest that the abnormal expression of AIF-1 may be linked to the immune response in cancers, which could offer insights for further exploration of the functions and roles of AIF-1 in the onset and progression of cancer.

### AIF-1 promotes the ability of cell proliferation and invasion

**Figure 11:**
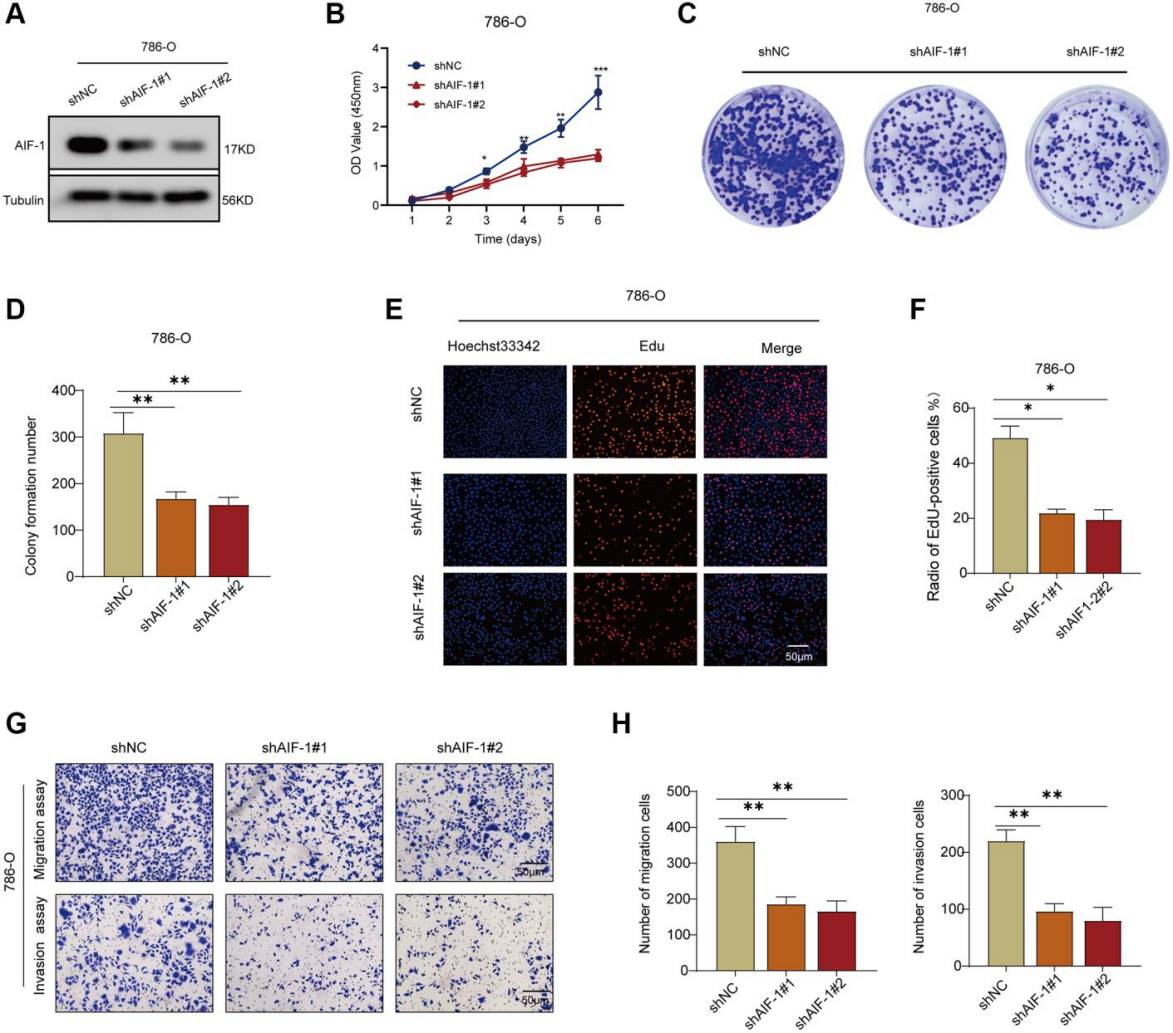
**AIF-1 promotes RCC cell proliferation and invasion.** (**A**) The protein expression levels of AIF-1 in 786-O cells after transfection with shAIF-1 or shNC. (**B**) CCK-8 assay showing proliferation of cells following knockdown of AIF-1. (**C** and **D**) Representative images and quantification of colony formation assays of 786-O cells transfected with shAIF-1. (**E** and **F**) Representative images and quantification of EdU assays of 786-O cells transfected with shAIF-1. Scale bar, 50 μm. (**G** and **H**) Transwell assays showed the suppressed migration and invasion ability of 786-O cells transfected with shAIF-1. (^*^P < 0.05, ^**^P < 0.01, and ^***^P < 0.001).

The following assays were designed to investigate the impact of AIF-1 on the proliferation and invasion of clear cell renal cells. First, western blotting was conducted to verify the knockdown status of AIF-1 in the 786-O cells. The Western blot analysis demonstrated that using sh-1 and sh-2 shRNA effectively reduced the protein level of AIF-1 in 786-O cells (Figure 11A). To investigate the possible role of AIF-1 in regulating cell proliferation and invasion, we conducted colony formation and Transwell assays to evaluate the proliferative and invasive capabilities of the cells. CCK-8 (Figure 11B) and colony formation (Figure 11C and 11D) analyses suggested that the proliferative ability of 786-O cells decreased after AIF-1 was knocked down. Edu experiment attained the same results (Figure 11E and 11F). Furthermore, the Transwell assays provided evidence that suppressing the expression of AIF-1 resulted in a notable decrease in the invasive capacity of 786-O cells. (Figure 11G and 11H). Therefore, we believe that AIF-1 expression plays a crucial role in promoting the proliferation and invasion of clear-cell renal cells.

## DISCUSSION

The analysis of gene expression through transcriptome analysis presents the most significant potential to investigate the intricate and diverse nature of different cancers and to pinpoint novel prognostic and therapeutic biomarkers. A substantial body of research has linked the expression of AIF-1 to cancer progression, metastasis, and prognosis, indicating that AIF-1 could serve as a valuable biomarker and therapeutic target. Consequently, it is essential to evaluate the role of AIF-1 across various cancer types thoroughly. In this study, we extensively examined AIF-1 expression levels in multiple databases, covering a wide range of cancers. Our analysis revealed that AIF-1 exhibited a significant upregulation in 17 types of tumor tissues compared to their normal tissue counterparts. It was found to be closely associated with the prognosis of several cancer types.

Additionally, we further investigated the correlation between AIF-1 expression and gene mutations, gene modifications, immune cell infiltration, TMB, and MSI in different cancers. Of equal importance, we carried out a series of experiments to investigate the expression of AIF-1 in KIRC tissues and renal cell carcinoma (RCC) cells. Our results indicated that AIF-1 was differentially expressed in these samples. We further demonstrated that AIF-1 exerted a pro-tumorigenic effect in RCC cells.

In the current pan-cancer analysis, based on the TCGA and GTEx databases, the expression of AIF-1 was observed to be significantly upregulated in the majority of cancer types studied, including BRCA, CESC, ESCA, CHOL, GBM, HNSC, KIRC, LAML, LGG, LIHC, OV, PAAD, SKCM, STAD, TGCT, THCA, and UCS. We found lower expression of AIF-1 in ACC, KICH, LUAD, LUSC, READ, and UCEC compared with normal tissues. Previous reports have suggested that AIF-1 could be a valuable prognostic biomarker for patients with glioma [12]. To confirm the findings from the bioinformatic analyses, we carried out a clinical sample test using KIRC samples. Our results indicated that the expression of AIF-1 was significantly higher in KIRC tissues at both the mRNA and protein levels compared to the corresponding adjacent normal kidney tissue. Taken together, these findings indicate that AIF-1 is prominently upregulated and expressed in most cancers, which strongly implies that AIF-1 may play a crucial role in the initiation and progression of tumorigenesis. The genetic mutations in normal cells can lead to the progression from hyperplasia and dysplasia to invasive cancer and ultimately to metastatic disease [24]. Therefore, analysis of genetic alterations can provide further insights into cancer progression [25]. Thus, we explored the mutation landscape of AIF-1 across cancers.

The findings of this study demonstrated that alterations in AIF-1 were not ubiquitous, with the most frequently affected cancer type being lymphoid neoplasm diffuse large B-cell lymphoma (DLBC), with over 8% of DLBC patients exhibiting alterations, mainly in the form of deep deletions. Amplification was the most common type of mutation in most cancers. Given that genetic alterations are a common feature in tumors, alterations observed in the precancerous stages are more likely to be pivotal events that trigger and propel cancer development [24]. However, it is worth noting that cancer progression cannot be attributed to mutations in a single gene. The cooccurrence of mutations in multiple genes is often observed as a typical driver to promote tumor progression and limit treatment response [26, 27]. Accordingly, we analyzed gene expression and alteration cooccurrence to identify potential functional partners of AIF-1 in various cancer types. Intriguingly, we found a remarkable correlation between AIF-1 expression and the expression of HSPA1L, PRRC2A, C2, MICB, SLC44A4, AMSH5, VWA7, C4A, LTA, and NELFE in pan-cancer analysis, which also suggested that they have a certain relationship in the carcinogenic mechanism. Notably, our results indicate that patients without alterations in AIF-1 exhibited improved disease-free and progression-free survival compared to those with AIF-1 alterations. Nevertheless, additional experimental studies will be necessary to comprehensively elucidate the mechanisms by which AIF-1 mutations contribute to tumorigenesis and disease progression.

Tumor immune escape is closely related to the prognosis and treatment of cancer patients [28]. Tumor immune escape mechanisms include two types. Tumor infiltration of immune cells leads to T-cell anergy or dysfunction [29], and the abnormal infiltration of immune cells into healthy tissues may facilitate the development and progression of tumors [30]. The second mechanism is T-cell rejection [31], through which tumors prevent immune cell infiltration. This process relies on immunosuppressive cells, including CAFs, Tregs, and MDSCs [32]. Studies have also found that AIF-1 plays a major role in infiltrating immune cells and mediating tumor progression, implying its high potential as a target molecule for breast cancer diagnosis, prognostication, and treatment [33]. In esophageal cancer, AIF-1 has emerged as a novel prognostic gene related to the tumor microenvironment, immune infiltration, and TIGIT expression. These findings suggest that AIF-1 holds great promise as a prognostic predictor [34, 35].

Importantly, our study found that AIF-1 is positively associated with the degree of infiltration of CD8+ T cells, monocytes, and macrophages but negatively associated with the degree of infiltration of dendritic cells and MDSCs in most cancers. Therefore, we speculate that T-cell rejection is the main mechanism by which AIF-1 regulates immune cell tumor escape, tumor promotion, and metastasis. Correlation analysis between AIF-1 and immune regulators in various cancers revealed a positive association between AIF-1 expression and numerous immune regulator genes in most tumors, except for THYM. This suggests that AIF-1 may play a role in modulating the immune response in tumor microenvironments. Moreover, AIF-1 correlated with TMB in 12 cancers and MSI in 11 cancers, which suggested that AIF-1 can be used as a new biomarker to predict ICI response for certain cancers. In previous studies, targeting CD28 [36], CD84 [37], CD86 [38], and LAIRI [39]. Based on our findings that AIF-1 expression is positively correlated with the expression of CD28, CD84, CD86, and LAIRI in several cancer types, we hypothesize that further investigation into the potential association between AIF-1 and these immune regulators would be promising.

The results from OS and DSS analyses were highly consistent, showing that AIF-1 is significantly associated with the prognosis of cancer patients, and AIF-1 is a risk factor for LGG and UVM and a protective factor for CESC, SKCM, and THCA. Combined with the previous AIF-1 expression analysis, we determined that high expression of AIF-1 results in a worse prognosis in LGG and UVM. The findings of the OS and DSS analyses were extremely consistent, indicating that AIF-1 is significantly connected with cancer patient prognosis and that AIF-1 is a risk factor for LGG and UVM and a protective factor for CESC, SKCM, and THCA. Combined with the earlier AIF-1 expression investigation, we discovered that high AIF-1 expression results in a poor prognosis in LGG and UVM. Furthermore, the Kaplan-Meier survival curves for KIRC provided clear evidence of a correlation between elevated AIF-1 expression levels and unfavorable prognosis. An earlier investigation has confirmed that AIF-1 holds promise as a prognostic biomarker for individuals with glioma. AIF-1 participates in pro-tumoral processes, governs immune status, and correlates with unfavorable prognoses [12]. The findings above indicate that AIF-1 has a crucial role in predicting the prognosis of patients and has the potential to serve as a robust biomarker for predicting certain types of cancer.

Additionally, we found that AIF-1 is hypermethylated in CHOL, THCA, PRAD, UCEC, BRCA, LUSC, and LUAD. At the same time, it is hypomethylated in COAD, HNSC, TGCT, BLCA, and KIRC. There was a correlation between the varying methylation statuses of AIF-1 and differential mRNA overexpression levels in those cancer types, indicating that epigenetic methylation of AIF-1 might impact the transcriptome of diverse cancers. The methylation statuses of AIF-1 in TCGA tumors were found to be associated with the levels of mRNA overexpression in those tumors. Surprisingly, our findings revealed that hypomethylation of AIF-1 resulted in shorter life durations in GBMLGG, KIRC, OV, and UVM; however, hypermethylation of AIF-1 resulted in shorter life durations in UCEC and melanoma. As a result, epigenetic methylation of AIF-1 may impact the transcriptome of TCGA tumor cells, which could, in turn, influence the development and progression of tumors.

Remarkably, according to the GSEA findings, AIF-1 was closely linked with immune-activated processes such as TNFA-signaling-via NFKB, KARAS-signaling-up, IFN-a response, IFN-g response, inflammatory-response, and allograft-rejection pathways. Still, the results differed significantly among various cancer types. For instance, AIF-1 was significantly negatively correlated with MYC-targets-V2, MITOTIC-signaling, G2-checkpoint, and E2F-targets in COAD, ESCA, and STAD. This discovery suggests that AIF-1 may have different functions in different types of cancer. Research by YANG et al. also demonstrated that AIF-1 was an independent prognostic indicator that regulates the β-catenin signaling pathway in gastric cancer [40]. AIF-1^+^ CSF1R^+^ MSCs, induced by TNF-α, generate an inflammatory microenvironment and promote hepatocarcinogenesis [41]. Furthermore, the PAK5-AIF signaling pathway may play an essential role in mammary tumorigenesis, providing a new therapeutic target for breast cancer treatment [42].

In this pan-cancer study, we investigated various aspects of AIF-1 expression. We determined that AIF-1 has the potential as a valuable biomarker in diverse cancers, particularly in the age of immunotherapy. However, despite these promising findings, some limitations still need to be considered. First, the results of this pan-cancer analysis were mainly derived from an integrated analysis of multiple databases. Due to the limited analysis method, this study may have some systematic errors. Second, this study presents the role of AIF-1 in various cancers through bioinformatics analysis, is validated by clinical specimens from KIRC, and uses a renal cell carcinoma cell line to perform functional cell testing *in vitro*. Third, we found that the AIF-1 expression is associated with tumor immunity. However, the specific mechanism of action is still unclear and needs further exploration. Nevertheless, this pan-cancer study provides a deeper understanding of the role of AIF-1 in the functional nucleus of different tumors.

## CONCLUSION

Our in-depth analysis of AIF-1 across multiple cancer types has shown its potential as a biomarker for predicting cancer prognosis. In addition, our research revealed a significant correlation between AIF-1 expression and the tumor microenvironment, tumor-infiltrating immune cells, immune subtypes, and biomarkers of immune checkpoint inhibitors. These discoveries offer novel insights into the potential involvement of AIF-1 in tumor immunity, which could aid in the identification of innovative therapeutic targets and predictive biomarkers for immunotherapy. Moreover, our study established the first evidence of differential expression of AIF-1 in KIRC tissues. It explored the effects of AIF-1 on the proliferation and invasion of RCC cells, laying a preliminary groundwork for developing targeted therapies for KIRC based on AIF-1 as a potential biomarker.

## Supplementary Materials

Supplementary Figures

Supplementary Table 1

## References

[1] Siegel RL, Miller KD, Fuchs HE, Jemal A. Cancer Statistics, 2021. CA Cancer J Clin. 2021; 71:7–33. 10.3322/caac.21654 33433946

[2] Cancer Genome Atlas Research Network. Comprehensive genomic characterization defines human glioblastoma genes and core pathways. Nature. 2008; 455:1061–8. 10.1038/nature07385 18772890 PMC2671642

[3] Barretina J, Caponigro G, Stransky N, Venkatesan K, Margolin AA, Kim S, Wilson CJ, Lehár J, Kryukov GV, Sonkin D, Reddy A, Liu M, Murray L, et al. Addendum: The Cancer Cell Line Encyclopedia enables predictive modelling of anticancer drug sensitivity. Nature. 2019; 565:E5–6. 10.1038/s41586-018-0722-x 30559381

[4] Hutter C, Zenklusen JC. The Cancer Genome Atlas: Creating Lasting Value beyond Its Data. Cell. 2018; 173:283–5. 10.1016/j.cell.2018.03.042 29625045

[5] Blum A, Wang P, Zenklusen JC. SnapShot: TCGA-Analyzed Tumors. Cell. 2018; 173:530. 10.1016/j.cell.2018.03.059 29625059

[6] Akbani R, Ng PK, Werner HM, Shahmoradgoli M, Zhang F, Ju Z, Liu W, Yang JY, Yoshihara K, Li J, Ling S, Seviour EG, Ram PT, et al. A pan-cancer proteomic perspective on The Cancer Genome Atlas. Nat Commun. 2014; 5:3887. 10.1038/ncomms4887 24871328 PMC4109726

[7] Weinstein JN, Collisson EA, Mills GB, Shaw KR, Ozenberger BA, Ellrott K, Shmulevich I, Sander C, Stuart JM, and Cancer Genome Atlas Research Network. The Cancer Genome Atlas Pan-Cancer analysis project. Nat Genet. 2013; 45:1113–20. 10.1038/ng.2764 24071849 PMC3919969

[8] Sikora M, Kopeć B, Piotrowska K, Pawlik A. Role of allograft inflammatory factor-1 in pathogenesis of diseases. Immunol Lett. 2020; 218:1–4. 10.1016/j.imlet.2019.12.002 31830499

[9] Autieri MV, Kelemen S, Thomas BA, Feller ED, Goldman BI, Eisen HJ. Allograft inflammatory factor-1 expression correlates with cardiac rejection and development of cardiac allograft vasculopathy. Circulation. 2002; 106:2218–23. 10.1161/01.cir.0000035652.71915.00 12390951

[10] Kimura M, Kawahito Y, Obayashi H, Ohta M, Hara H, Adachi T, Tokunaga D, Hojo T, Hamaguchi M, Omoto A, Ishino H, Wada M, Kohno M, et al. A critical role for allograft inflammatory factor-1 in the pathogenesis of rheumatoid arthritis. J Immunol. 2007; 178:3316–22. 10.4049/jimmunol.178.5.3316 17312183

[11] Jia J, Bai Y, Fu K, Sun ZJ, Chen XM, Zhao YF. Expression of allograft inflammatory factor-1 and CD68 in haemangioma: implication in the progression of haemangioma. Br J Dermatol. 2008; 159:811–9. 10.1111/j.1365-2133.2008.08744.x 18647307

[12] Rao M, Yang Z, Huang K, Liu W, Chai Y. Correlation of AIF-1 Expression with Immune and Clinical Features in 1270 Glioma Samples. J Mol Neurosci. 2022; 72:420–32. 10.1007/s12031-021-01948-x 34939148

[13] Deininger MH, Seid K, Engel S, Meyermann R, Schluesener HJ. Allograft inflammatory factor-1 defines a distinct subset of infiltrating macrophages/microglial cells in rat and human gliomas. Acta Neuropathol. 2000; 100:673–80. 10.1007/s004010000233 11078219

[14] Liu S, Tan WY, Chen QR, Chen XP, Fu K, Zhao YY, Chen ZW. Daintain/AIF-1 promotes breast cancer proliferation via activation of the NF-kappaB/cyclin D1 pathway and facilitates tumor growth. Cancer Sci. 2008; 99:952–7. 10.1111/j.1349-7006.2008.00787.x 18341653 PMC11159275

[15] Li T, Feng Z, Jia S, Wang W, Du Z, Chen N, Chen Z. Daintain/AIF-1 promotes breast cancer cell migration by up-regulated TNF-α via activate p38 MAPK signaling pathway. Breast Cancer Res Treat. 2012; 131:891–8. 10.1007/s10549-011-1519-x 21509525

[16] Scott AJ, Wilkinson AS, Wilkinson JC. Basal metabolic state governs AIF-dependent growth support in pancreatic cancer cells. BMC Cancer. 2016; 16:286. 10.1186/s12885-016-2320-3 27108222 PMC4841948

[17] Jia S, Du Z, Jiang H, Huang X, Chen Z, Chen N. Daintain/AIF-1 accelerates the activation of insulin-like growth factor-1 receptor signaling pathway in HepG2 cells. Oncol Rep. 2015; 34:511–7. 10.3892/or.2015.4002 25998745

[18] Cerami E, Gao J, Dogrusoz U, Gross BE, Sumer SO, Aksoy BA, Jacobsen A, Byrne CJ, Heuer ML, Larsson E, Antipin Y, Reva B, Goldberg AP, et al. The cBio cancer genomics portal: an open platform for exploring multidimensional cancer genomics data. Cancer Discov. 2012; 2:401–4. 10.1158/2159-8290.CD-12-0095 22588877 PMC3956037

[19] Sun D, Wang J, Han Y, Dong X, Ge J, Zheng R, Shi X, Wang B, Li Z, Ren P, Sun L, Yan Y, Zhang P, et al. TISCH: a comprehensive web resource enabling interactive single-cell transcriptome visualization of tumor microenvironment. Nucleic Acids Res. 2021; 49:D1420–30. 10.1093/nar/gkaa1020 33179754 PMC7778907

[20] Franz M, Rodriguez H, Lopes C, Zuberi K, Montojo J, Bader GD, Morris Q. GeneMANIA update 2018. Nucleic Acids Res. 2018; 46:W60–4. 10.1093/nar/gky311 29912392 PMC6030815

[21] Yu G, Wang LG, Han Y, He QY. clusterProfiler: an R package for comparing biological themes among gene clusters. OMICS. 2012; 16:284–7. 10.1089/omi.2011.0118 22455463 PMC3339379

[22] Greillier L, Tomasini P, Barlesi F. The clinical utility of tumor mutational burden in non-small cell lung cancer. Transl Lung Cancer Res. 2018; 7:639–46. 10.21037/tlcr.2018.10.08 30505708 PMC6249623

[23] Hause RJ, Pritchard CC, Shendure J, Salipante SJ. Classification and characterization of microsatellite instability across 18 cancer types. Nat Med. 2016; 22:1342–50. 10.1038/nm.4191 27694933

[24] Garnis C, Buys TP, Lam WL. Genetic alteration and gene expression modulation during cancer progression. Mol Cancer. 2004; 3:9. 10.1186/1476-4598-3-9 15035667 PMC408463

[25] Hahn WC, Weinberg RA. Rules for making human tumor cells. N Engl J Med. 2002; 347:1593–603. 10.1056/NEJMra021902 12432047

[26] Blakely CM, Watkins TBK, Wu W, Gini B, Chabon JJ, McCoach CE, McGranahan N, Wilson GA, Birkbak NJ, Olivas VR, Rotow J, Maynard A, Wang V, et al. Evolution and clinical impact of co-occurring genetic alterations in advanced-stage EGFR-mutant lung cancers. Nat Genet. 2017; 49:1693–704. 10.1038/ng.3990 29106415 PMC5709185

[27] Hong S, Gao F, Fu S, Wang Y, Fang W, Huang Y, Zhang L. Concomitant Genetic Alterations With Response to Treatment and Epidermal Growth Factor Receptor Tyrosine Kinase Inhibitors in Patients With EGFR-Mutant Advanced Non-Small Cell Lung Cancer. JAMA Oncol. 2018; 4:739–42. 10.1001/jamaoncol.2018.0049 29596544 PMC5885210

[28] Lawal B, Lin LC, Lee JC, Chen JH, Bekaii-Saab TS, Wu ATH, Ho CL. Multi-Omics Data Analysis of Gene Expressions and Alterations, Cancer-Associated Fibroblast and Immune Infiltrations, Reveals the Onco-Immune Prognostic Relevance of STAT3/CDK2/4/6 in Human Malignancies. Cancers (Basel). 2021; 13:954. 10.3390/cancers13050954 33668805 PMC7956610

[29] Yu GP, Chiang D, Song SJ, Hoyte EG, Huang J, Vanishsarn C, Nadeau KC. Regulatory T cell dysfunction in subjects with common variable immunodeficiency complicated by autoimmune disease. Clin Immunol. 2009; 131:240–53. 10.1016/j.clim.2008.12.006 19162554 PMC5140037

[30] Man YG, Stojadinovic A, Mason J, Avital I, Bilchik A, Bruecher B, Protic M, Nissan A, Izadjoo M, Zhang X, Jewett A. Tumor-infiltrating immune cells promoting tumor invasion and metastasis: existing theories. J Cancer. 2013; 4:84–95. 10.7150/jca.5482 23386907 PMC3564249

[31] Joyce JA, Fearon DT. T cell exclusion, immune privilege, and the tumor microenvironment. Science. 2015; 348:74–80. 10.1126/science.aaa6204 25838376

[32] Komohara Y, Fujiwara Y, Ohnishi K, Takeya M. Tumor-associated macrophages: Potential therapeutic targets for anti-cancer therapy. Adv Drug Deliv Rev. 2016; 99:180–5. 10.1016/j.addr.2015.11.009 26621196

[33] Slim FA, Ouellette G, Ennour-Idrissi K, Jacob S, Diorio C, Durocher F. An isoform of AIF1 involved in breast cancer. Cancer Cell Int. 2018; 18:167. 10.1186/s12935-018-0663-3 30386176 PMC6198497

[34] Xu X, Wang D, Li N, Sheng J, Xie M, Zhou Z, Cheng G, Fan Y. The Novel Tumor Microenvironment-Related Prognostic Gene AIF1 May Influence Immune Infiltrates and is Correlated with TIGIT in Esophageal Cancer. Ann Surg Oncol. 2022; 29:2930–40. 10.1245/s10434-021-10928-9 34751872

[35] Xu X, Wang D, Li N, Sheng J, Xie M, Zhou Z, Cheng G, Fan Y. ASO Author Reflection: The TME-Related Gene AIF1 Signature Predicts Esophageal Carcinoma Prognosis. Ann Surg Oncol. 2022; 29:2941. 10.1245/s10434-021-10955-6 35169974

[36] Khan M, Arooj S, Wang H. Soluble B7-CD28 Family Inhibitory Immune Checkpoint Proteins and Anti-Cancer Immunotherapy. Front Immunol. 2021; 12:651634. 10.3389/fimmu.2021.651634 34531847 PMC8438243

[37] Lewinsky H, Gunes EG, David K, Radomir L, Kramer MP, Pellegrino B, Perpinial M, Chen J, He TF, Mansour AG, Teng KY, Bhattacharya S, Caserta E, et al. CD84 is a regulator of the immunosuppressive microenvironment in multiple myeloma. JCI Insight. 2021; 6:141683. 10.1172/jci.insight.141683 33465053 PMC7934939

[38] Wennhold K, Thelen M, Lehmann J, Schran S, Preugszat E, Garcia-Marquez M, Lechner A, Shimabukuro-Vornhagen A, Ercanoglu MS, Klein F, Thangarajah F, Eidt S, Löser H, et al. CD86^+^ Antigen-Presenting B Cells Are Increased in Cancer, Localize in Tertiary Lymphoid Structures, and Induce Specific T-cell Responses. Cancer Immunol Res. 2021; 9:1098–108. 10.1158/2326-6066.CIR-20-0949 34155067

[39] Ramos MIP, Tian L, de Ruiter EJ, Song C, Paucarmayta A, Singh A, Elshof E, Vijver SV, Shaik J, Bosiacki J, Cusumano Z, Jensen C, Willumsen N, et al. Cancer immunotherapy by NC410, a LAIR-2 Fc protein blocking human LAIR-collagen interaction. Elife. 2021; 10:e62927. 10.7554/eLife.62927 34121658 PMC8225389

[40] Ye Y, Miao S, Lu R, Xia X, Chen Y, Zhang J, Wu X, He S, Qiang F, Zhou J. Allograft inflammatory factor-1 is an independent prognostic indicator that regulates β-catenin in gastric cancer. Oncol Rep. 2014; 31:828–34. 10.3892/or.2013.2915 24337893

[41] Zong C, Meng Y, Ye F, Yang X, Li R, Jiang J, Zhao Q, Gao L, Han Z, Wei L. AIF1^+^ CSF1R^+^ MSCs, induced by TNF-α, act to generate an inflammatory microenvironment and promote hepatocarcinogenesis. Hepatology. 2022. [Epub ahead of print]. 10.1002/hep.32738 35989499 PMC10344441

[42] Xing Y, Li Y, Hu B, Han F, Zhao X, Zhang H, Li Y, Li D, Li J, Jin F, Li F. PAK5-mediated AIF phosphorylation inhibits its nuclear translocation and promotes breast cancer tumorigenesis. Int J Biol Sci. 2021; 17:1315–27. 10.7150/ijbs.58102 33867848 PMC8040471

