# Supplementary material for: Allograft inflammatory factor 1 is a potential diagnostic, immunological, and prognostic biomarker in pan-cancer
Source: Aging (Albany NY). 2023 Apr 3;15(7):2582–609. doi: 10.18632/aging.204631 (PMC10120906; doi:10.18632/aging.204631)
Supplement: Supplementary Figures [file aging-15-204631-s001.pdf]

SUPPLEMENTARY FIGURES

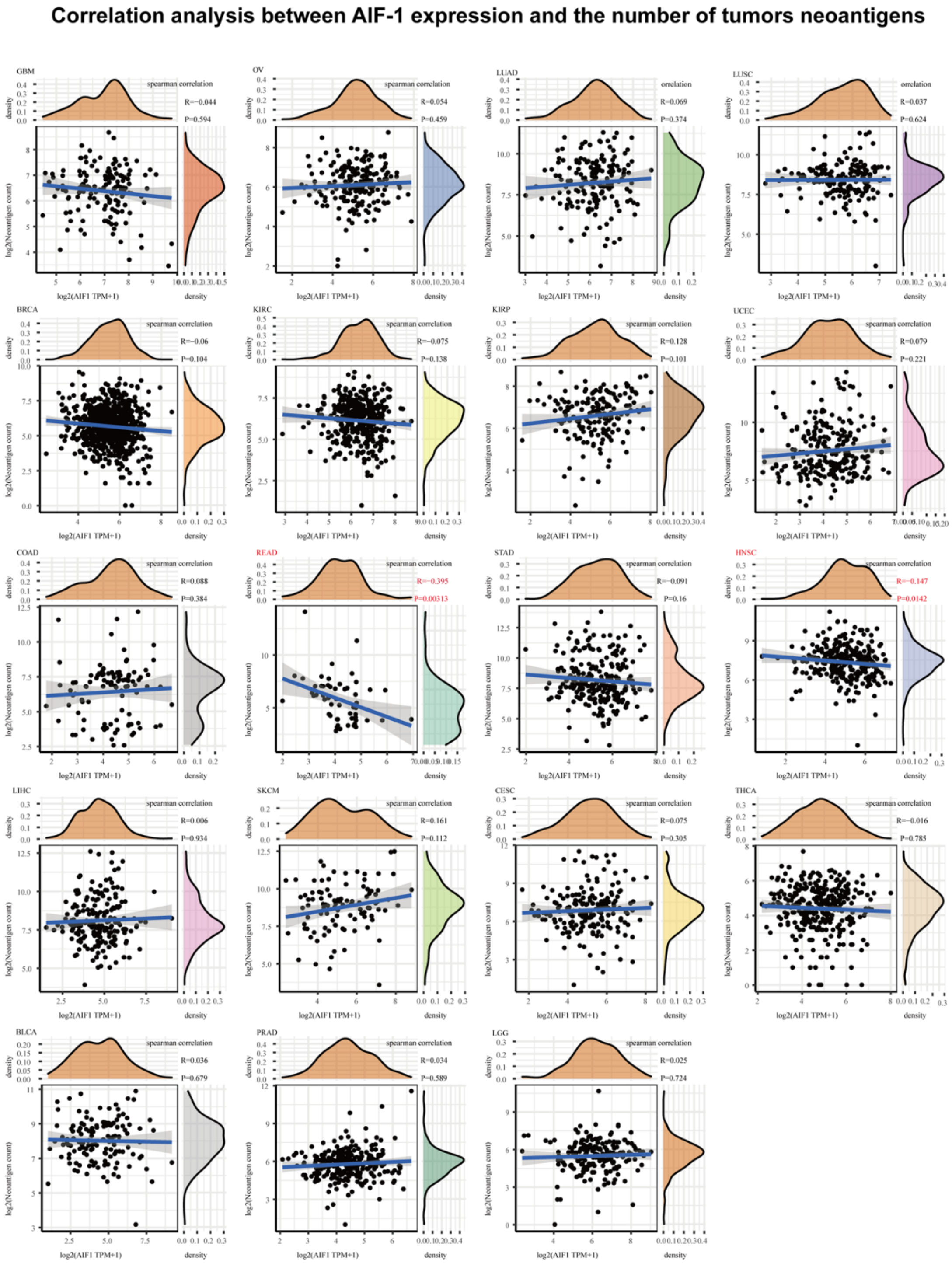

Supplementary Figure 1. Correlation analysis between AIF-1 expression across cancers and the number of tumors neoantigens.

Relationship between AIF-1 expression and the StromalScores.

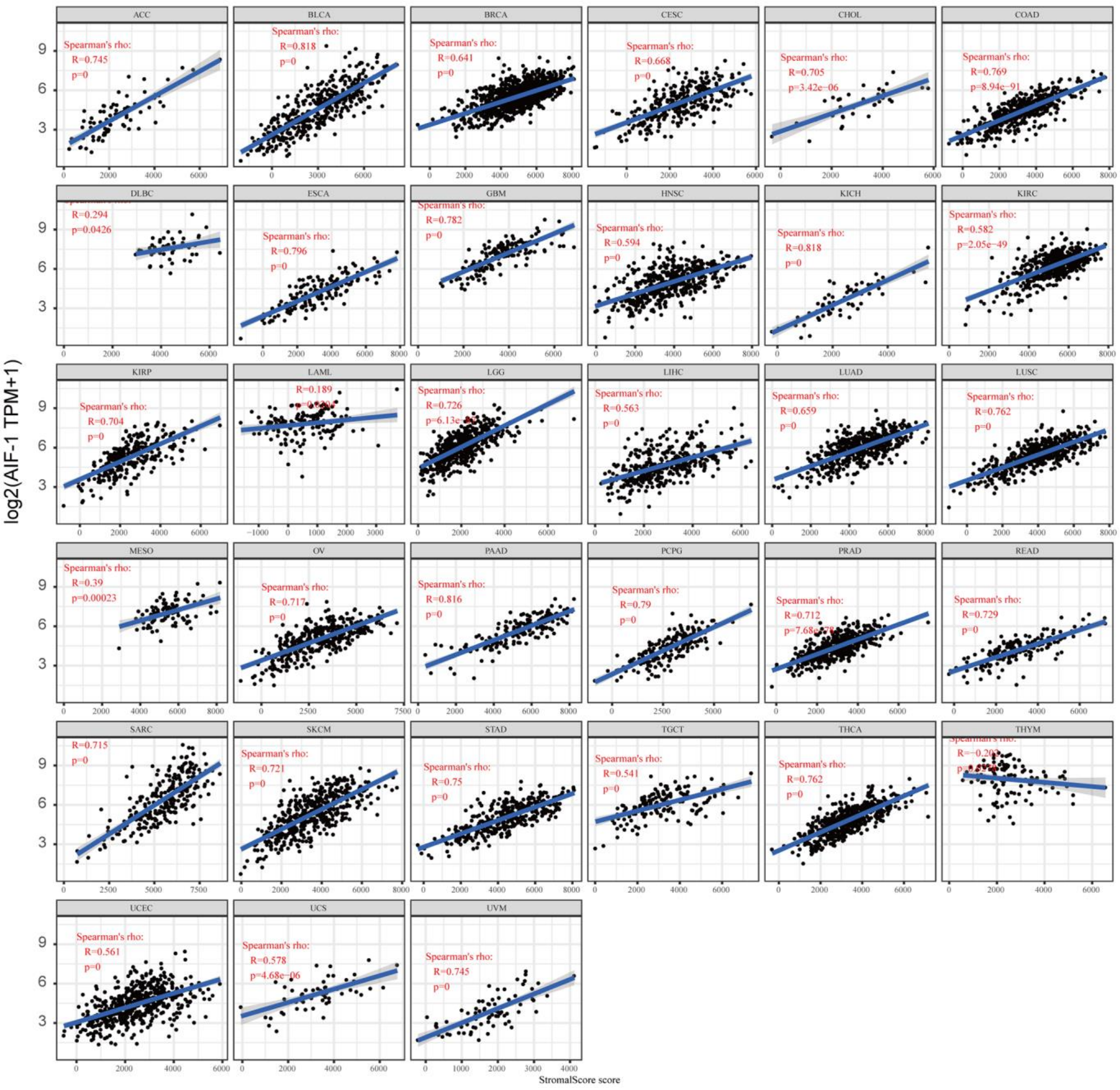

Supplementary Figure 2. Correlation analysis between the expression levels of AIF-1 and the StromalScore across cancers.

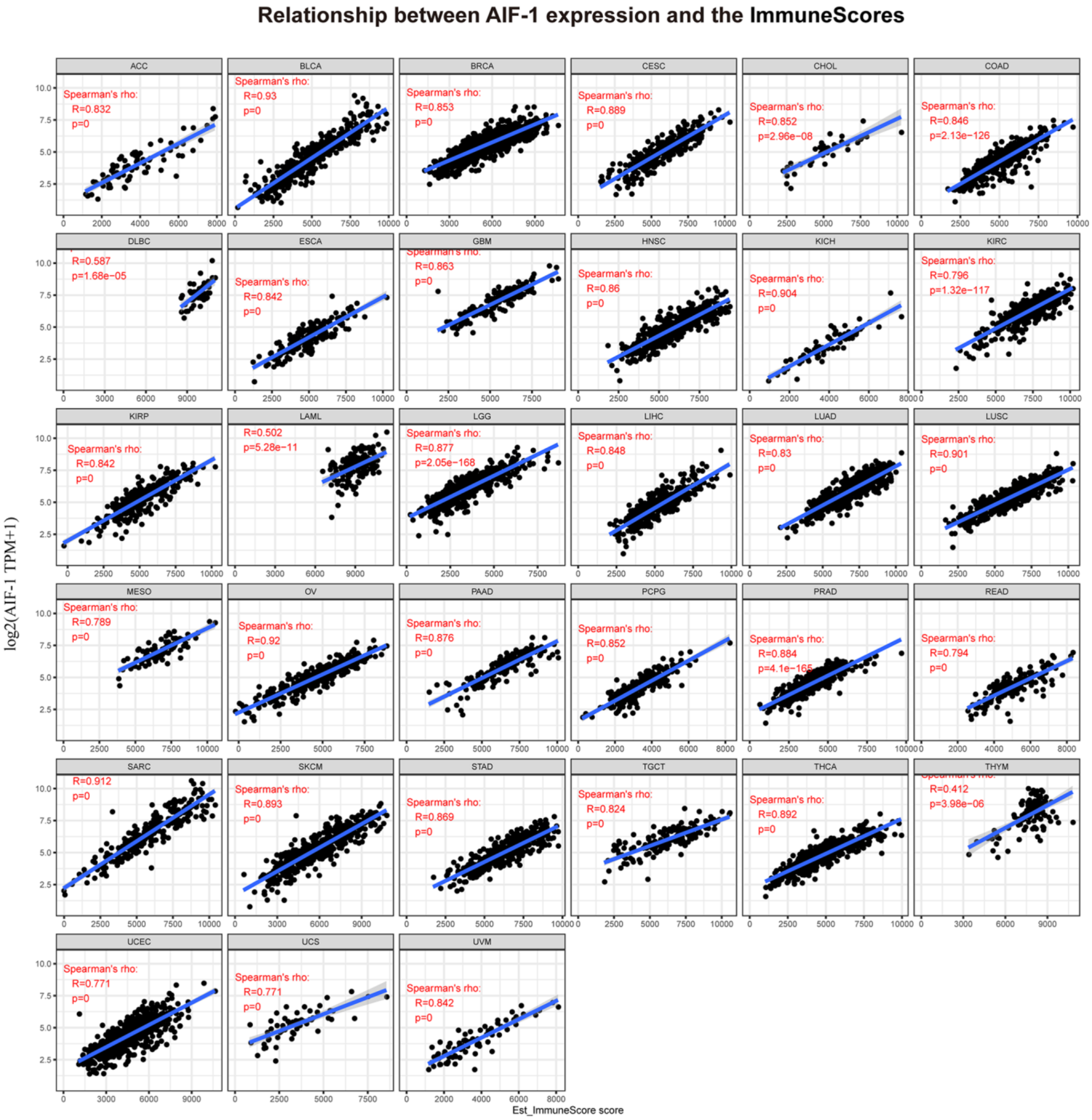

Supplementary Figure 3. Correlation analysis between the expression levels of AIF-1 and the ImmuneScore across cancers.

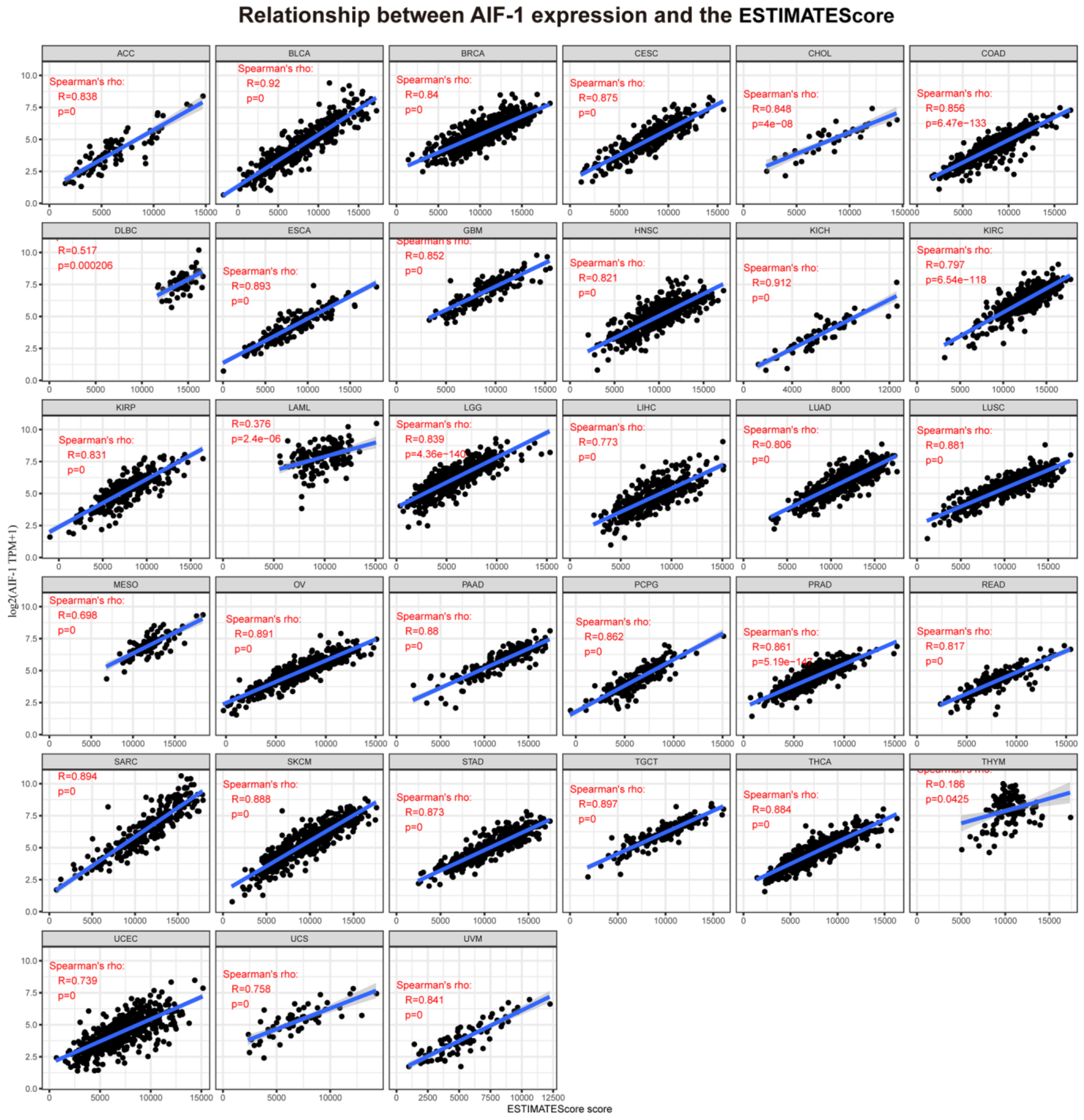

Supplementary Figure 4. Correlation analysis between the expression levels of AIF-1 and EstimateScore across cancers.

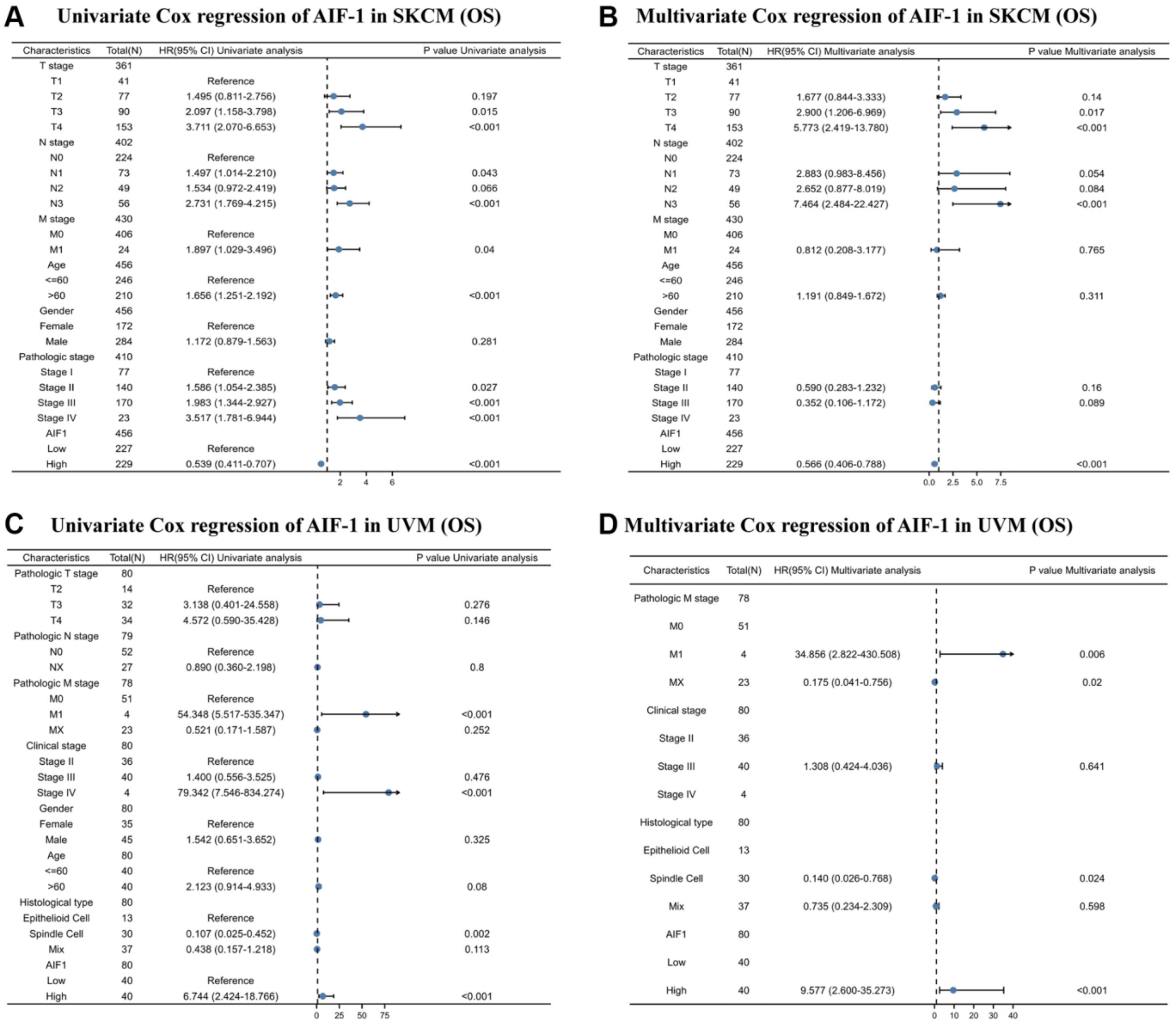

Supplementary Figure 5. Prognostic value of AIF-1 expression. (**A**) Univariate Cox regression of AIF-1 in SKCM (OS). (**B**) Multivariate Cox regression of AIF-1 in SKCM (OS). (**C**) Univariate Cox regression of AIF-1 in UVM (OS). (**D**) Multivariate Cox regression of AIF-1 in UVM (OS).
